# Comparative Diagnostic and Prognostic Performance of SWI and T2-Weighted MRI in Cerebral Microbleed Detection Following Acute Ischemic Stroke: A Meta-Analysis and SPOT-CMB Study

**DOI:** 10.3390/medicina61091566

**Published:** 2025-08-30

**Authors:** Rachel Tan, Kevin J. Spring, Murray Killingsworth, Sonu Bhaskar

**Affiliations:** 1 Global Health Neurology Lab, Sydney, NSW 2150, Australia; 2 UNSW Medicine and Health, South Western Sydney Clinical Campuses, University of New South Wales (UNSW), Sydney, NSW 2052, Australia; 3 Clinical Sciences Stream, Ingham Institute for Applied Medical Research, Sydney, NSW 2170, Australia; 4 NSW Brain Clot Bank, NSW Health Pathology, Sydney, NSW 2170, Australia; 5 Medical Oncology Group, Ingham Institute for Applied Medical Research, Sydney, NSW 2751, Australia; 6 School of Medicine, Western Sydney University, Sydney, NSW 2000, Australia; 7 Correlative Microscopy Facility, Department of Anatomical Pathology, NSW Health Pathology, and Liverpool Hospital, Liverpool, NSW 2170, Australia; 8 Department of Neurology & Neurophysiology, Liverpool Hospital & South Western Sydney Local Health District (SWSLHD), Sydney, NSW 2170, Australia; 9 Department of Neurology, Division of Cerebrovascular Medicine & Neurology, National Cerebral and Cardiovascular Center (NCVC), Suita 564-8565, Osaka, Japan

**Keywords:** cerebral microbleeds, acute ischemic stroke, susceptibility-weighted imaging, T2*-weighted imaging, symptomatic intracerebral hemorrhage, hemorrhagic transformation, functional decline

## Abstract

*Background and Objectives*: Cerebral microbleeds (CMBs) are increasingly being considered as potential biomarkers of small vessel disease and cerebral vulnerability, particularly in patients with acute ischemic stroke (AIS). Accurate detection is crucial for prognosis and therapeutic decision-making, yet the relative utility of susceptibility-weighted imaging (SWI) versus T2*-weighted imaging (T2*) remains uncertain. Materials and Methods: We conducted a systematic review and meta-analysis (SPOT-CMB, *Susceptibility-weighted imaging and Prognostic Outcomes in Acute Stroke—Cerebral Microbleeds* study) of 80 studies involving 28,383 AIS patients. Pooled prevalence of CMBs was estimated across imaging modalities (SWI, T2*, and both), and stratified analyses examined variation by demographic, clinical, and imaging parameters. Meta-analytic odds ratios assessed associations between CMB presence and key outcomes: symptomatic intracerebral hemorrhage (sICH), hemorrhagic transformation (HT), and poor functional outcome (modified Rankin Scale score 3–6) at 90 days. Diagnostic performance was assessed using summary receiver operating characteristic curves. *Results*: Pooled CMB prevalence was higher with SWI (36%; 95% CI 31–41) than T2* (25%; 95% CI 22–28). CMB presence was associated with increased odds of sICH (OR 2.22; 95% CI 1.56–3.16), HT (OR 1.33; 95% CI 1.01–1.75), and poor 90-day outcome (OR 1.61; 95% CI 1.39–1.86). However, prognostic performance was modest, with low sensitivity (e.g., AUC for sICH: 0.29) and low diagnostic odds ratios. SWI outperformed T2* in detection but offered limited prognostic gain. Access to SWI remains limited in many settings, posing challenges for global implementation. *Conclusions*: SWI detects CMBs more frequently than T2* in AIS patients and shows stronger associations with adverse outcomes, supporting its value for risk stratification. However, prognostic accuracy remains limited, and our GRADE appraisal indicated only moderate certainty for functional outcomes, with lower certainty for diagnostic accuracy due to heterogeneity and imprecision. These findings highlight the clinical utility of SWI but underscore the need for standardized imaging protocols and high-quality prospective studies.

## 1. Background

Cerebral microbleeds (CMBs) are small, chronic brain hemorrhages that are increasingly recognized as critical markers of cerebrovascular pathology, particularly following acute ischemic stroke (AIS) [1]. Globally, AIS remains a leading cause of mortality and morbidity, accounting for over 63.5 million disability-adjusted life years (DALYs) in 2019 [2]. The prevalence of CMBs rises from 10% in the general population to 34% in AIS patients, with higher rates observed in hypertensive and elderly individuals [3]. Accurate CMB detection is essential for guiding clinical decision-making, particularly for managing risks such as symptomatic intracerebral hemorrhage (sICH), hemorrhagic transformation (HT), stroke recurrence, and functional decline [4,5,6]. For instance, CMB presence can influence the initiation of anticoagulation therapy or other therapeutic interventions, underscoring its prognostic significance [7]. Beyond being static markers of past hemorrhage, emerging evidence suggests that CMBs may represent dynamic sites of ongoing microvascular injury, inflammation, and impaired glymphatic clearance, a concept we refer to as the *Living Lesion Paradigm* (under review) [8]. This paradigm has important implications for both imaging-based detection and clinical outcomes in AIS patients, warranting a re-evaluation of CMBs as active contributors to disease progression rather than inert remnants [8].

Currently, CMB detection relies on magnetic resonance imaging (MRI) sequences, with hemosiderin-sensitive techniques such as T2*-weighted imaging (T2*) and susceptibility-weighted imaging (SWI) being the most effective [9]. T2* is widely used for its accessibility but has limited sensitivity [10], while SWI offers superior contrast and sensitivity, though it is not yet standard in many acute stroke protocols [11]. In contrast, other imaging modalities, such as fluid-attenuated inversion recovery (FLAIR) and non-contrast computed tomography (NCCT), are less reliable for CMB detection due to poor sensitivity to blood degradation products [12,13], although their effectiveness when combined with SWI or T2* remains unclear. Despite these advancements, there remains a limited understanding of the extent to which SWI offers additional diagnostic benefit over T2*. Additionally, the lack of standardized imaging protocols and variability in reported diagnostic accuracy across modalities pose significant challenges for clinical implementation [14]. Addressing these gaps, our systematic review and meta-analysis (SPOT-CMB, *Susceptibility-weighted imaging and Prognostic Outcomes in Acute Stroke—Cerebral Microbleeds* study) aimed to systematically evaluate CMB prevalence based on the use of SWI or T2*, quantify their relative detection performance, and explore the clinical implications of CMB burden in AIS patients.

The primary objective of this study was to undertake a comprehensive evaluation of CMBs in the context of AIS. Our objectives were to (1) estimate the pooled prevalence of CMBs in AIS patients using SWI compared with T2*; (2) assess how prevalence varies according to patient demographics, stroke subtype, imaging parameters, and regional differences; and (3) evaluate the association of CMBs with clinically relevant outcomes, including sICH, HT, and functional outcome at 90 days.

## 2. Materials and Methods

### 2.1. Literature Search and Study Selection

**Figure 1:**
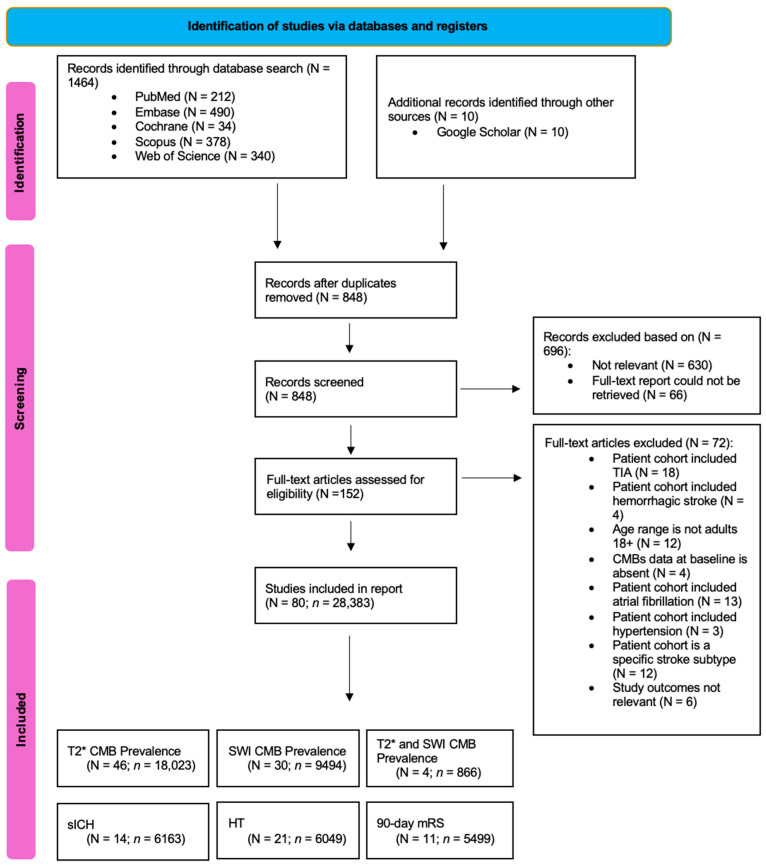
PRISMA Flowchart: Inclusion of Studies in the Meta-Analysis. Illustration depicting the flow of study selection according to the PRISMA guidelines, leading to the inclusion of studies in the meta-analysis. Abbreviations: CMB = cerebral microbleed, N = number of studies, *n* = cohort size, TIA = transient ischemic attack, T2* = T2 Gradient Echo Imaging, SWI = Susceptibility Weighted Imaging, sICH = symptomatic intracranial hemorrhage, HT = hemorrhagic transformation, mRS = Modified Rankin Scale.

A comprehensive literature search was conducted using PubMed, EMBASE, the Cochrane Central Register of Controlled Trials, Scopus, and Web of Science to identify relevant studies published between January 2000 and May 2025. The search strategy incorporated a combination of keywords, including “cerebral microbleeds”, “microhemorrhages”, “ischemic stroke”, “cerebral infarction”, “susceptibility-weighted imaging”, “T2-star imaging”, “flair attenuated inversion recovery”, or “non-contrast computed tomography”. The full search strategy is provided in the Online Supplementary Information (Search Strategy). In addition to database searches, the reference lists of relevant studies, systematic reviews, and meta-analyses were screened to capture any additional eligible studies. The study selection process, inclusion criteria, and subgroup analyses conducted as part of this meta-analysis were summarized using a PRISMA (Preferred Reporting Items for Systematic Reviews and Meta-Analyses) flow diagram (Figure 1). This review was conducted following the PRISMA 2020 guidelines (Supplemental Table S1) and the MOOSE (Meta-analysis of Observational Studies in Epidemiology) reporting standards (Supplemental Table S2), as detailed in the Supplementary Information. This study was registered in Open Science, registration number “fks6z” (https://osf.io/fks6z/, accessed on 19 July 2025).

### 2.2. Inclusion and Exclusion Criteria

Studies were deemed eligible for inclusion if they met the following criteria: (a) patients diagnosed with AIS; (b) patients aged 18 years or older; (c) reported data on the baseline presence of CMBs; (d) availability of comparative data between CMB-positive and CMB-negative groups for relevant post-stroke outcomes; (e) applied an appropriate study design with a minimum sample size of at least 20 patients.

Studies were excluded if they were: (a) systematic reviews, meta-analyses, case reports, or narrative reviews; (b) involved animal experiments; (c) did not provide access to full-text articles; (d) lacked relevant data on baseline CMB status or post-stroke outcomes; (e) were not published in English; (f) duplicated publications.

### 2.3. Data Extraction

All article titles and abstracts were initially screened using EndNote (Clarivate Analytics, London, UK) to exclude studies that did not meet the predefined eligibility criteria. Full-text articles for potentially relevant studies were then assessed in detail to determine final eligibility for inclusion in the systematic review and meta-analysis. Data extraction was performed using a standardized data collection form, which captured the following information from each study:

(1) Study characteristics: author, country, publication year, study name or registry, study design, cohort size;
(2) Participant characteristics: age, sex, comorbidities, number of patients with CMBs at baseline, stroke subtype, CMB location, and specific characteristics of patients with AIS;
(3) Imaging parameters: MRI sequence type for CMB detection, field strength, slice thickness.
  The ‘SWI and T2*’ subgroup is defined as studies that visualized CMBs in their patients using either SWI or T2* sequences. Slice thickness was extracted as reported and categorized using study-defined thresholds: Thin (≤2 mm), Medium (2.1–4.9 mm), and Thick (≥5 mm), based on radiological conventions commonly applied in neuroimaging studies [15,16];
(4) Definition and criteria of various parameters: CMBs, sICH, poor functional outcome;
(5) Clinical outcomes: occurrence of sICH, HT, and mRS score for functional outcome at 90 days, assessed in relation to the presence or absence of CMBs.

Disagreements were resolved through discussions, and if unresolved, adjudicated by a third reviewer.

### 2.4. Methodological Quality Assessment of Included Studies

The methodological quality of the included studies was assessed using the Modified Jadad Analysis (MJA) [17] (Supplemental Table S3). In addition, potential bias related to funding sources was examined by reviewing each study’s declarations of funding and conflicts of interest (Supplemental Table S4).

### 2.5. Certainty of Evidence Assessment

We evaluated the certainty of evidence using the Grading of Recommendations, Assessment, Development and Evaluations (GRADE) approach. Each outcome (symptomatic intracerebral hemorrhage, hemorrhagic transformation, and poor functional outcome) was assessed for study limitations (risk of bias), inconsistency, indirectness, imprecision, and publication bias. A Summary of Findings (SoF) table (SPOT-CMB GRADE SoF) was constructed, presenting pooled effect estimates, absolute effects, and certainty ratings. This allowed us to make a transparent comparison of strengths and limitations across outcomes and facilitated interpretation of the prognostic value of CMBs.

### 2.6. Statistical Analyses

All statistical analyses were conducted using STATA version 13.0 (StataCorp, College Station, TX, USA). Baseline characteristics of the included study populations were extracted, with means and standard deviations (SDs) estimated from medians and interquartile ranges (IQRs) when necessary, following the method proposed by Wan et al. [18].

The pooled prevalence of CMBs among AIS patients across different imaging modalities was calculated using the “metaprop” package, applying a random-effects meta-analysis for proportions derived from individual studies. Exact 95% confidence intervals (CIs) were generated using the “cimethod (exact”) and “ftt” commands. To investigate associations between CMB presence and clinical outcomes, random-effects meta-analyses were performed using the DerSimonian and Laird method. This analysis was restricted to studies reporting baseline CMB data and outcomes related to CMB presence or absence. The random-effects method was consistently applied across all subgroup analyses, which included comparisons based on the imaging modality used (SWI, T2*, or SWI and T2*).

Forest plots were generated to present pooled odds ratios (ORs), 95% CIs, and inter-study heterogeneity. Heterogeneity was assessed using the I^2^ statistic, with thresholds defined as follows: <30% indicating low heterogeneity, 30–50% moderate, 50–70% substantial, and >75% considerable heterogeneity. Cochran’s Q test *p* values and Tau-squared (τ^2^) were also reported to further quantify heterogeneity. Sensitivity analyses were conducted using the “metainf” package, assessing the influence of individual studies on the overall estimates by systematically excluding one study at a time.

Potential publication bias was evaluated using Egger’s regression test, visual inspection of funnel plots, and Deeks’ funnel plot asymmetry test, generated with the “metabias”, “metafunnel”, and “midas” packages, respectively. Publication bias was assessed for all primary outcomes (prevalence, sICH, HT, and functional outcome) using Egger’s test, funnel plots, and Deeks’ test. Asymmetry within the funnel plot, along with significant Egger’s test results, was considered indicative of publication bias. To evaluate diagnostic performance and the association between CMBs and clinical outcomes, the “midas’ package was used. Accuracy plots summarizing pooled estimates of sensitivity, specificity, likelihood ratios, diagnostic odds ratios (DORs), and other test performance metrics were generated using the midas command with the res(all) option. Summary Receiver Operating Characteristic (SROC) curves were constructed with 95% confidence and prediction contours using the “plot sroc(both)” function. Lastly, Fagan’s Nomogram was generated to illustrate the relationships among pre-test probability, likelihood ratios, and post-test probability, using the “midas” package. All statistical tests were two-sided, with significance set at *p* < 0.05.

## 3. Results

### 3.1. Description of Included Studies

A total of 1464 studies were initially identified through electronic database searches. After duplicate records were removed, 848 studies remained for screening. Following a detailed review of titles and abstracts, 696 studies were excluded based on relevance and inability to retrieve full-text reports. Of the 152 full-text articles assessed, 72 were excluded for multiple reasons: 22 studies included transient ischemic stroke (TIA) and/or hemorrhagic stroke in the patient cohort, 16 studies included atrial fibrillation and/or hypertension in the patient cohort, 12 studies had unsuitable age ranges, 4 studies were missing CMB data at baseline, 12 studies looked at a specific stroke subtype, and 6 studies did not report the primary outcomes of interest. Ultimately, 80 studies, comprising 28,383 patients, were included in this meta-analysis. Among these studies, 46 detected CMBs using T2* [19,20,21,22,23,24,25,26,27,28,29,30,31,32,33,34,35,36,37,38,39,40,41,42,43,44,45,46,47,48,49,50,51,52,53,54,55,56,57,58,59,60,61,62,63,64], 30 used SWI [65,66,67,68,69,70,71,72,73,74,75,76,77,78,79,80,81,82,83,84,85,86,87,88,89,90,91,92,93,94], and 4 used T2* and SWI [7,95,96,97].

Table 1, Table 2, and Table 3 present an overview of the clinical characteristics, risk factors, and outcomes of participants across the studies, respectively. Table 4 summarizes findings related to heterogeneity and estimated pooled prevalence of CMBs across different modalities and clinical parameters. Additional insight into the association between CMBs and prognostic outcomes such as sICH, HT, and mRS scores at 90 days is presented in Table 5, while Table 6 presents information on diagnostic and prognostic performance. It is important to note that variations in the definitions of CMBs and sICH existed across the studies.

A comprehensive evaluation of methodological quality and funding bias is presented in Supplemental Table S3 and Supplemental Table S4. Assessment of publication bias using Egger’s test revealed no significant evidence of small-study effects across key outcomes, as illustrated in Supplemental Table S5. Finally, Supplemental Table S6 presents the results from Deeks’ test, providing further assessment of publication bias.

### 3.2. Prevalence of CMBs Using Different Imaging Modalities

**Figure 2:**
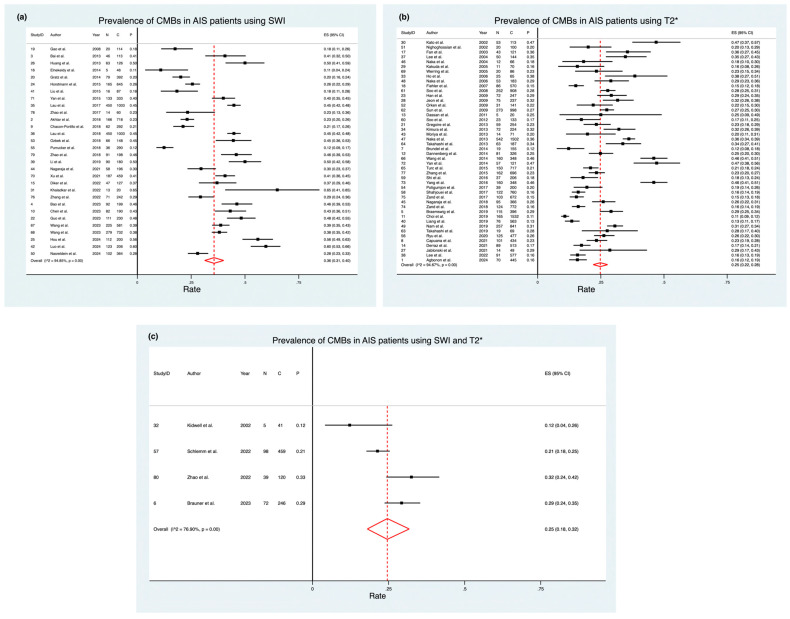
Forest Plots: Pooled Prevalence of CMBs based on Different Imaging Modalities [7,19,20,21,22,23,24,25,26,27,28,29,30,31,32,33,34,35,36,37,38,39,40,41,42,43,44,45,46,47,48,49,50,51,52,53,54,55,56,57,58,59,60,61,62,63,64,65,66,67,68,69,70,71,72,73,74,75,76,77,78,79,80,81,82,83,84,85,86,87,88,89,90,91,92,93,94,95,96,97]. (**a**) Prevalence of CMBs in AIS patients assess using SWI. (**b**) Prevalence of CMBs in AIS patients assessed using T2*. (**c**) Prevalence of CMBs in AIS patients assesed using both SWI and T2*. The red diamonds indicate the pooled prevalence estimates with 95% confidence intervals, and the red dashed vertical lines represent the overall pooled prevalence across studies. Abbreviations: CMB = cerebral microbleed, CI = confidence interval, T2* = T2 Gradient Echo Imaging, SWI = Susceptibility Weighted Imaging, N = number of patients, C = cohort size, P = prevalence, AIS = acute ischemic stroke, ES = effect size.

A comprehensive analysis of all 80 studies (*n* = 28,383) revealed the overall pooled prevalence of CMBs to be 29% (95% CI: 0.26; 0.31) in AIS patients. Subgroup analysis based on the type of modality used revealed that patients undergoing SWI for CMB detection [65,66,67,68,69,70,71,72,73,74,75,76,77,78,79,80,81,82,83,84,85,86,87,88,89,90,91,92,93,94] had the highest pooled prevalence, at 36% (95% CI: 0.31; 0.41) (Figure 2). Studies that used T2* [19,20,21,22,23,24,25,26,27,28,29,30,31,32,33,34,35,36,37,38,39,40,41,42,43,44,45,46,47,48,49,50,51,52,53,54,55,56,57,58,59,60,61,62,63,64] had a prevalence of 25% (95% CI: 0.22; 0.28), while those that used SWI and T2* [7,95,96,97] exhibited a prevalence of 25% (95% CI; 0.18; 0.32) (Figure 2). Notably, significant heterogeneity persisted within these subgroups (I^2^ = 95.87%, *p* < 0.001), with a heterogeneity chi^2^ of 1912.84 (*p* < 0.001, d.f. 79). The high heterogeneity observed for pooled prevalence estimates (I^2^ > 90%) reflected methodological and population variability across the 80 included studies. In contrast, lower heterogeneity values in outcome analyses (e.g., sICH I^2^ = 29.7%, mRS I^2^ = 0%) arose from smaller and more clinically homogeneous subsets of studies. Sensitivity analyses confirmed that no single study drove the pooled estimates, though heterogeneity persisted in prevalence analyses. While formal meta-regression was limited by data availability, subgroup analyses (by age, hypertension, imaging parameters) partially reduced heterogeneity, supporting these factors as potential contributors. Overall, these results highlight the influence of detection methods on reported CMB burden.

#### 3.2.1. Stratified by Age

Age-related differences were examined in this meta-analysis. For SWI-detected CMBs, 13 studies (*n* = 1436) [65,70,72,73,78,79,80,81,82,83,85,86,92] assessed pooled CMB prevalence in adults aged 65 and older, while 6 studies (*n* = 673) [67,68,75,76,89,90] focused on those under 65. The meta-analysis indicated an estimated pooled prevalence of 35% (95% CI: 0.28; 0.43) in the older group, and 36% (95% CI: 0.27; 0.47) in the younger group.

For T2*-weighted MRI, 24 studies (*n* = 2893) [21,24,28,29,32,33,35,37,38,39,40,43,44,47,48,52,54,55,56,58,59,60,62,63] examined adults 65 and older, and 10 (*n* = 787) [20,22,25,30,31,46,49,50,51,64] focused on those under 65. The corresponding pooled prevalence was 25% (95% CI: 0.21; 0.30) and 22% (95% CI: 0.18; 0.26), respectively. These results align with established evidence that advancing age is a major determinant of small vessel pathology and higher CMB prevalence, consistent with population-based imaging studies [98]. Significant heterogeneity was observed for both modalities, with a chi^2^ of 377.01 (*p* < 0.001, d.f. 18) for SWI and 779.72 (*p* < 0.001, d.f. 33) for T2*.

#### 3.2.2. Stratified by Hypertension Rates

Given the potential role of vascular risk factors in CMB development, we next examined hypertension’s influence. In studies using SWI, 14 studies (*n* = 1980) [65,66,68,75,76,77,78,79,81,85,86,89,90,92] assessed CMB prevalence in cohorts with an average hypertension rate of 65% or higher, while 5 studies (*n* = 486) [70,80,82,83,88] focused on cohorts with rates below 65%. The meta-analysis indicated an estimated pooled prevalence of 36% (95% CI: 0.29; 0.43) in the higher hypertension group and 37% (95% CI: 0.27; 0.48) in the lower hypertension group.

For studies using T2*, 15 (*n* = 1631) [19,21,22,29,32,33,35,42,46,47,56,57,58,59,64] assessed cohorts with hypertension rates of 65% or higher, and 12 (*n* = 893) [20,24,25,30,45,48,49,52,55,60,62,63] focused on rates below 65%. The corresponding pooled prevalence was 26% (95% CI: 0.0.23; 0.29) and 21% (95% CI: 0.17; 0.27), respectively. This gradient in prevalence highlights the well-established association between hypertension and the development of microangiopathic changes that underlie CMBs [1]. Significant heterogeneity was observed for both modalities, with a chi^2^ of 418.37 (*p* < 0.001, d.f. 18) for SWI and 300.82 (*p* < 0.001, d.f. 26) for T2*.

#### 3.2.3. Stratified by Regional Variation

Geographic disparities were then analyzed to reflect differences in population-specific risk factors. Twenty-nine studies assessed regional differences in CMB prevalence among AIS patients using SWI. In European cohorts, 5 studies (*n* = 393) [70,71,78,79,83] had a pooled prevalence of 27% (95% CI: 0.18; 0.37), while in Asian cohorts, 20 studies (*n* = 2820) [65,66,67,68,72,73,75,77,80,81,82,84,86,87,88,89,90,91,92,93] had a pooled prevalence of 41% (95% CI: 0.37; 0.46). In North America, 2 studies (*n* = 120) [76,85] had a pooled prevalence of 24% (95% CI: 0.21; 0.28), while in Africa, 2 studies (*n* = 107) [69,94] had a pooled prevalence of 26% (95% CI: 0.22; 0.30).

Forty-six studies assessed regional differences in CMB prevalence among AIS patients using T2*. In European cohorts, 13 studies (*n* = 774) [20,25,32,34,36,41,42,45,53,59,60,61,63] had a pooled prevalence of 21% (95% CI: 0.19; 0.24), while in Asian cohorts, 27 studies (*n* = 2970) [19,21,22,23,26,27,29,30,31,33,35,37,38,39,40,43,44,46,47,48,49,54,55,56,57,58,62] had a pooled prevalence of 28% (95% CI: 0.19; 0.24). In North America, 4 studies (*n* = 444) [50,51,52,64] had a pooled prevalence of 18% (95% CI: 0.14; 0.22), while 2 multinational studies (*n* = 97) [24,28] had a pooled prevalence of 15% (95% CI: 0.12; 0.18). Significant heterogeneity was observed between regions, with a chi^2^ of 559.19 (*p* < 0.001, d.f. 28) for SWI and 844.41 (*p* < 0.001, d.f. 45) for T2*. The consistently higher prevalence observed in Asian cohorts may reflect differences in genetic susceptibility, vascular risk profiles, and lifestyle factors. These findings underscore the importance of considering population-level variation when interpreting CMB burden.

#### 3.2.4. Stratified by Use of FLAIR

The addition of FLAIR sequences to SWI protocols were analyzed, where 17 studies (*n* = 2090) [52,65,66,67,73,75,77,79,81,83,84,86,87,88,89,93,94] assessed the estimated pooled prevalence of CMBs amongst AIS patients when using SWI and FLAIR, while 10 studies (*n* = 846) [68,69,70,71,72,76,78,80,82,92] estimated pooled prevalence when SWI was used but not FLAIR. The meta-analysis revealed an estimated pooled prevalence of 38% (95% CI: 0.31; 0.44) when FLAIR was added, and 33% (95% CI: 0.25; 0.42) when FLAIR was not added.

Twenty eight (28) studies (*n* = 2131) [19,24,25,26,27,29,30,32,35,36,38,40,41,43,44,45,46,47,50,51,52,55,57,58,59,61,62,64] assessed the estimated pooled prevalence of CMBs amongst AIS patients when using T2* and FLAIR, while 18 studies (*n* = 2154) [20,21,22,23,28,31,33,34,37,39,42,48,49,53,54,56,60,63] estimated pooled prevalence when T2* was used but not FLAIR. The meta-analysis revealed an estimated pooled prevalence of 24% (95% CI: 0.21; 0.27) when FLAIR was added, and 26% (95% CI: 0.20; 0.31) when FLAIR was not added. Significant heterogeneity was observed, with a chi^2^ of 555.50 (*p* < 0.001, d.f. 26) for SWI and 844.41 (*p* < 0.001, d.f. 45) for T2*.

#### 3.2.5. Stratified by Use of NCCT

The effect of incorporating NCCT with SWI protocols was analyzed, where 6 studies (*n* = 509) [67,78,81,88,93,94] examined the estimated pooled prevalence of CMBs amongst AIS patients when using SWI and NCCT, while 21 studies (*n* = 2427) [65,66,68,69,70,71,72,73,75,76,77,79,80,82,83,84,85,86,87,89,92] estimated pooled prevalence when SWI was used but not NCCT. The meta-analysis revealed an estimated pooled prevalence of 44% (95% CI: 0.21; 0.33) when NCCT was used, and 24% (95% CI: 0.21; 0.26) when NCCT was not used. While intriguing, this discrepancy likely reflects the very small number of studies in the NCCT+SWI subgroup and should not be overinterpreted. NCCT is generally insensitive to CMBs, and further validation is required before firm conclusions can be drawn [98].

Sixteen studies (*n* = 2194) [20,21,26,28,29,30,33,37,38,39,43,48,52,54,62,64] examined the estimated pooled prevalence of CMBs amongst AIS patients when using T2* and NCCT, while 30 studies (*n* = 2091) [19,22,23,24,25,27,31,32,34,35,36,40,41,42,44,45,46,47,49,50,51,53,55,56,57,58,59,60,61,63] estimated pooled prevalence when T2* was used but not NCCT. The meta-analysis revealed an estimated pooled prevalence of 27% (95% CI: 0.21; 0.33) when NCCT was added, and 24% (95% CI: 0.21; 0.26) when NCCT was not added. Significant heterogeneity was observed, with a chi^2^ of 555.50 (*p* < 0.001, d.f. 26) for SWI and 844.41 (*p* < 0.001, d.f. 45) for T2*.

#### 3.2.6. Stratified by Use of Slice Thickness

The effect of scanner parameters, such as slice thickness, was explored. Eleven studies (*n* = 712) [44,66,68,72,78,80,81,84,86,93,94] assessed pooled CMB prevalence when using thin slices, 4 studies (*n* = 326) [69,71,85,95] looked at medium slices, and 33 studies (*n* = 3397) [7,20,21,22,23,24,26,27,28,29,30,31,33,34,35,36,37,39,41,42,43,45,46,47,49,52,53,60,62,73,76,77,79] examined thick slices.

A comparison between SWI and T2* was unable to be made due to a lack of studies within the slice thickness subgroups. The meta-analysis revealed an estimated pooled prevalence of 40% (95% CI: 0.32; 0.49) for thin slices, 23% (95% CI: 0.18; 0.28) for medium slices, and 25% (95% CI: 0.22; 0.29) for thick slices. These results reinforced prior imaging studies showing that thinner slices increase lesion detectability and should be adopted as standard where feasible to optimize CMB detection [15]. The chi^2^ for heterogeneity was 809.02 (*p* < 0.001, d.f. 47).

#### 3.2.7. Stratified by Field Strength

Field strength was another parameter that influenced CMB prevalence. Fourteen studies (*n* = 2048) [65,66,68,71,73,75,76,77,78,80,82,84,86,89] examined the estimated pooled prevalence of CMBs amongst AIS patients when using SWI at 3 Tesla, while 8 studies (*n* = 521) [67,69,72,81,83,87,93,94] estimated pooled prevalence when SWI was used at a lower field strength of 1.5 Tesla. The meta-analysis revealed an estimated pooled prevalence of 37% (95% CI: 0.31; 0.43) at 3 Tesla, and 36% (95% CI: 0.26; 0.47) at 1.5 Tesla.

Nine studies (*n* = 927) [30,42,44,46,50,51,53,62,64] examined the estimated pooled prevalence of CMBs amongst AIS patients when using T2* at 3 Tesla, while 22 studies (*n* = 2074) [19,20,21,22,24,25,29,31,32,33,34,35,36,38,39,40,43,45,49,55,59,61] estimated pooled prevalence when T2* was used at a lower field strength of 1.5 Tesla. The meta-analysis revealed an estimated pooled prevalence of 23% (95% CI: 0.18; 0.28) at 3 Tesla and 27% (95% CI: 0.23; 0.31) at 1.5 Tesla. While counterintuitive, these findings may reflect small subgroup sizes and methodological inconsistencies across studies rather than the true superiority of 1.5T. Larger, harmonized datasets are needed to clarify the relationship between field strength and CMB detection. Significant heterogeneity was observed, with a chi^2^ of 370.43 (*p* < 0.001, d.f. 21) for SWI and 460.20 (*p* < 0.001, d.f. 30) for T2*.

#### 3.2.8. Stratified by Stroke Subtype

Stroke subtype was another important factor associated with variation in prevalence estimates. Using SWI, 5 studies (*n* = 129) [54,66,75,85,89] assessed pooled CMB prevalence in patients with an atherothrombotic stroke subtype, 5 studies (*n* = 168) [54,66,75,85,89] focused on lacunar stroke, 5 (*n* = 159) [54,66,75,85,89] on cardio-embolism, and 4 (*n* = 61) [54,66,85,89] on undetermined stroke subtypes. The meta-analysis indicated an estimated pooled prevalence of 23% (95% CI: 0.08; 0.42) in atherothrombotic stroke, 26% (95% CI: 0.17; 0.37) in lacunar stroke, 25% (95% CI: 0.11; 0.43) in cardio-embolism, and 20% (95% CI: 0.10; 0.32) in undetermined stroke subtype.

Using T2*, 5 studies (*n* = 218) [19,23,33,43,59] assessed pooled CMB prevalence in patients with an atherothrombotic stroke subtype, 5 studies focused on lacunar stroke (*n* = 206) [19,23,33,43,59], 5 (*n* = 67) [19,23,33,43,59] on cardio-embolism, and 3 (*n* = 66) [33,43,59] on undetermined stroke subtypes. The meta-analysis indicated an estimated pooled prevalence of 25% (95% CI: 0.12; 0.39) in atherothrombotic stroke, 39% (95% CI: 0.25; 0.53) in lacunar stroke, 24% (95% CI: 0.14; 0.35) in cardio-embolism, and 27% (95% CI: 0.20; 0.33) in undetermined stroke subtype. Significant heterogeneity was observed, with a chi^2^ of 229.98 (*p* < 0.001, d.f. 18) for SWI and 119.90 (*p* < 0.001, d.f. 17) for T2*.

#### 3.2.9. Stratified by CMB Location

The anatomical location of CMBs also contributed to variability in prevalence estimates. Using SWI, 11 studies (*n* = 549) [65,68,70,73,77,79,80,83,87,89,90] assessed pooled CMB prevalence in lobar locations, 9 (*n* = 172) [65,68,73,77,79,80,83,87,89] studies focused on infratentorial locations, 9 (*n* = 240) [65,68,73,77,79,80,83,87,89] on deep locations, and 10 (*n* = 816) [65,68,70,73,77,79,80,83,89,90] on mixed locations. The meta-analysis indicated an estimated pooled prevalence of 29% (95% CI: 0.24; 0.24) in lobar regions, 12% (95% CI: 0.07; 0.19) in infratentorial regions, 18% (95% CI: 0.14; 0.21) in deep regions, and 49% (95% CI: 0.39; 0.60) in patients with mixed CMB locations.

Using T2*, 12 studies (*n* = 419) [31,35,44,45,46,47,49,50,53,59,60,64] assessed pooled CMB prevalence in lobar locations, 5 (*n* = 19) [31,35,44,47,49] studies focused on infratentorial locations, 7 (*n* = 200) [31,35,44,46,47,49,64] on deep locations, and 11 (*n* = 476) [31,35,44,45,46,47,49,50,53,59,60] on mixed locations. The meta-analysis indicated an estimated pooled prevalence of 37% (95% CI: 0.29; 0.46) in lobar regions, 8% (95% CI: 0.02; 0.19) in infratentorial regions, 33% (95% CI: 0.20; 0.47) in deep regions, and 46% (95% CI: 0.36; 0.55) in patients with mixed CMB locations. Significant heterogeneity was observed, with a chi^2^ of 1021.49 (*p* < 0.001, d.f. 38) for SWI and 446 (*p* < 0.001, d.f. 34) for T2*.

### 3.3. Association of CMBs with Prognostic Outcomes

Table 5 summarizes the association between various prognostic outcomes and CMB prevalence in patients with AIS, while Table 6 presents information on diagnostic and prognostic performance. For more detailed information on these associations, refer to the supplemental figures (Supplemental Figures S4–S9), which provide information on publication bias and sensitivity analyses.

#### 3.3.1. Symptomatic Intracranial Hemorrhage (sICH)

**Figure 3:**
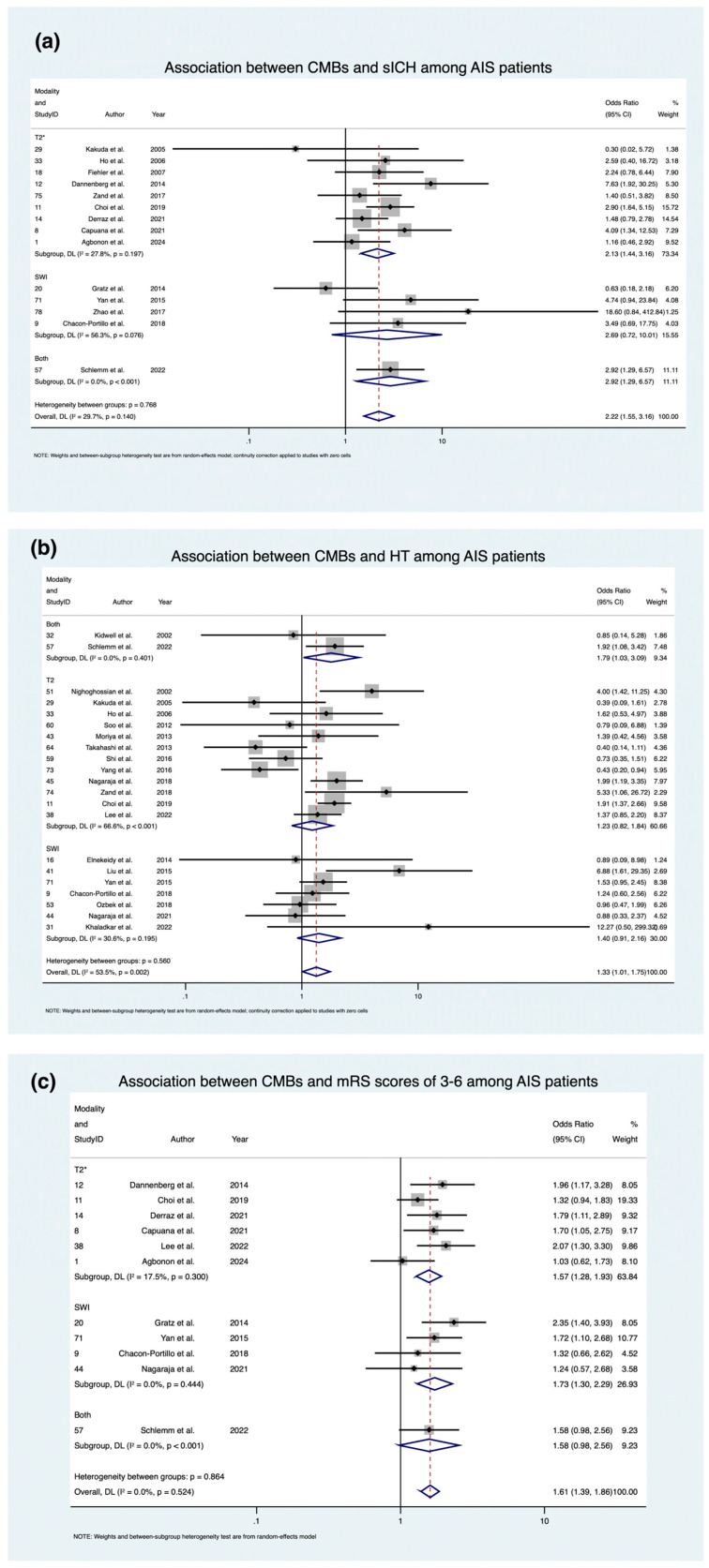
Forest Plots: Prognostic Outcome Analysis of CMBs Stratified by Imaging Modality [7,20,24,26,28,35,38,40,42,47,48,51,52,54,59,60,62,63,64,69,70,72,73,74,76,78,84,85,95]. (**a**) Association between CMBs and sICH among AIS patients. (**b**) Association between CMBs and HT among AIS patients. (**c**) Association between CMBs and poor functional outcome, defined as mRS scores of 3-6, among AIS patients. The blue diamonds represent the pooled odds ratios with 95% confidence intervals within each subgroup and for the overall analysis. Abbreviations: CMB = cerebral microbleed, sICH = symptomatic intracerebral hemorrhage, HT = hemorrhagic transformation, mRS = modified Rankin Score, AIS = acute ischemic stroke, CI = confidence interval, DL = DerSimonian and Laird method.

To explore the prognostic implications of CMBs in AIS, the association with sICH, a critical complication following stroke, was analyzed. The meta-analysis included a total of 14 studies, comprising 6163 patients, sub-grouped as follows: 4 studies [70,73,74,76] using SWI, 9 studies [24,26,28,42,51,54,59,60,63] using T2*, and 1 [95] employing both SWI and T2* for CMB detection, as evident in Figure 3. Various criteria were used to define sICH (Table 3), which introduced some heterogeneity in outcome reporting. CMB presence was associated with an overall increased risk of sICH, with an OR of 2.216 (95% CI: 1.555; 3.159, *p* < 0.0001). This trend was more pronounced when SWI and T2* were used to detect CMBs, presenting an OR of 2.916 (1.294; 6.574, *p* = 0.010). However, this relied on a limited dataset from a single study, warranting cautious interpretation. Similarly, within the subgroups of patients who received SWI and those that received T2* for CMB detection, there were increased odds of sICH (SWI: OR 2.687; CI: 0.722; 10.007, *p* = 0.141; T2*: OR 2.13; CI: 1.435; 3.160 s), but only T2* and studies that used SWI and T2* obtained statistical significance.

The overall heterogeneity of the meta-analysis was low, with an I^2^ of 29.7%, which was not statistically significant (*p* = 0.140). Visual inspection of the funnel plot revealed slight asymmetry (Supplemental Figure S5); however, Egger’s regression test showed no statistically significant evidence of small-study effects (*p* = 0.656) (Supplemental Figure S4). Similarly, Deeks’ test demonstrated no significant evidence of publication bias (*p* = 0.97) (Supplemental Figure S8).

Further sensitivity analyses included Fagan’s Nomogram, which demonstrated a weakly positive likelihood ratio of 2 (Supplemental Figure S9), and the SROC curve, which showed poor diagnostic performance with an AUC of 0.29 (Supplemental Figure S7). These findings are consistent with the influence analysis (Supplemental Figure S6) and the diagnostic performance summary stratified by imaging modality (Table 6). Specifically, SWI demonstrated a sensitivity of 0.05, specificity of 0.98, and a DOR of 3, while T2* showed a sensitivity of 0.09, specificity of 0.96, and a DOR of 2. These findings confirmed that while CMBs increase the risk of sICH (OR 2.216), their diagnostic performance remains limited, as reflected in the low AUC (0.29). Thus, CMBs should be interpreted as contributory risk markers rather than standalone predictors of post-stroke hemorrhage.

#### 3.3.2. Hemorrhagic Transformation (HT)

HT represents another important complication in AIS patients. The meta-analysis incorporated 21 studies, encompassing a total of 6049 patients, which were divided into subgroups based on imaging modality: 7 studies [69,72,73,76,78,84,85] using SWI, 12 studies [20,24,26,35,38,40,47,48,52,54,62,64] using T2*, and 2 studies [7,95] implementing both SWI and T2* for CMB detection (Figure 3). Definitions of HT were not always specified and varied across studies. The presence of CMBs was linked to a significantly higher risk of HT, with an OR of 1.332 (95% CI: 1.013; 1.750, *p* = 0.040). This association appeared more pronounced in the subset of studies using both SWI and T2*, which demonstrated an OR of 1.788 (95% CI: 1.033; 3.094, *p* = 0.038). Within the modality-specific subgroups, increased odds of HT were also observed for both SWI (OR 1.402; 95% CI: 0.910; 2.163, *p* = 0.125) and T2* (OR 1.229; 95% CI: 0.820; 1.843, *p* = 0.319), though statistical significance was achieved only for the combined SWI and T2* group.

Heterogeneity across studies was moderate, with an I^2^ statistic of 53.5% and a significant *p*-value (*p* = 0.002). Visual inspection of the funnel plot revealed minor asymmetry, suggestive of possible small-study effects (Supplemental Figure S5); however, Egger’s regression and Deeks’ test did not indicate significant publication bias (Supplemental Tables S5 and S6).

Additional sensitivity analyses were performed, including Fagan’s Nomogram (Supplemental Figure S9) and the SROC plot, with an AUC value of 0.56 (Supplemental Figure S7). These results were consistent with findings from the diagnostic performance summary (Supplemental Table S6). SWI demonstrated a sensitivity of 0.34, specificity of 0.75, and a DOR of 2, while T2* showed a sensitivity of 0.21, specificity of 0.82, and a DOR of 1. The pooled analysis demonstrated a significant association between CMBs and HT (OR 1.332), most evident in the combined SWI/T2* subgroup. However, moderate heterogeneity (I^2^ = 53.5%) reflected variability in population characteristics and imaging protocols, consistent with prior HT meta-analyses.

#### 3.3.3. mRS 3-6 at 90 Days

The presence of CMBs was also evaluated for its impact on long-term functional outcomes, specifically, disability at 90 days post-stroke, as measured by the mRS. The meta-analysis included a total of 11 studies involving 5499 patients, which were divided into subgroups based on imaging modality: 4 studies [70,73,76,85] using SWI, 6 studies [42,54,59,60,62,63] using T2*, and 1 study [95] implementing both SWI and T2* for CMB detection (Figure 3). All studies consistently defined poor functional outcome at 90 days as an mRS score between 3 to 6. Overall, the presence of CMBs was associated with significantly increased odds of poor functional outcome at 90 days (OR 1.606; 95% CI: 1.387; 1.858, *p* < 0.0001). This association appeared more pronounced in the subset of studies using SWI, which demonstrated an OR of 1.727 (95% CI: 1.303; 2.289, *p* < 0.0001). This was followed by the SWI and T2* combined subgroup (OR 1.727; 95% CI: 0.976; 2.555, *p* = 0.063), although caution is warranted as it relied on a limited data set of 1 study. Increased odds were also observed for the T2* subgroup (OR 1.572; 95% CI: 1.282; 1.927, *p* < 0.0001), though statistical significance was achieved only for studies that used SWI or T2*.

No heterogeneity existed across the included studies, with an I^2^ statistic of 0% and a *p*-value of 0.524. Visual inspection of the funnel plot revealed reasonable symmetry (Supplemental Figure S5), with Egger’s regression and Deeks’ test not indicating significant publication bias (Supplemental Tables S5 and S6).

Additional sensitivity analyses were performed, including Fagan’s Nomogram (Supplemental Figure S9) and the SROC plot, with an AUC value of 0.58 (Supplemental Figure S7). These results were consistent with findings from the diagnostic performance summary (Supplemental Table S6), which reported an overall sensitivity of 0.49, specificity of 0.62, and a DOR of 2. Subgroup analysis by imaging modality using the “midas” model could not be performed due to the small number of included studies and high variability. Nonetheless, the association with poor 90-day outcome (OR 1.606) was strikingly consistent, with no heterogeneity (I^2^ = 0%). This underscores CMBs as reliable prognostic indicators of post-stroke disability, representing the most robust outcome signal observed in this meta-analysis.

### 3.4. Methodological Quality

The Modified Jadad Scores (Supplemental Table S3) indicated moderate methodological quality across the included studies, with most scoring between 3.5 and 5.5. Variability in study design, blinding, and reporting likely contributed to the heterogeneity observed in the pooled estimates. These limitations highlight the need for higher-quality, standardized research to improve the reliability of future meta-analyses.

## 4. Discussion

This meta-analysis is distinct in terms of providing pooled prevalence estimates of CMBs in AIS patients based on different imaging modalities, with additional analyses exploring how prevalence varies by patient demographics, stroke subtypes, and clinical settings. Moreover, the meta-analysis also identifies that CMBs are associated with significantly increased odds of sICH, HT, and mRS scores, highlighting the clinical relevance of CMB detection in AIS. To our knowledge, no previous meta-analyses have directly compared SWI and T2* for detecting CMBs in AIS patients.

To further contextualize these findings, we summarized the certainty of evidence for each outcome using the GRADE approach (see Table 7). The findings suggest that while CMBs are consistently associated with increased risk of sICH, HT, and poor functional outcomes, the certainty of evidence is limited by observational design, heterogeneity in definitions, and imprecision. The GRADE SoF table provides a transparent appraisal of where evidence is more robust (e.g., poor functional outcome with moderate certainty) versus where conclusions should be interpreted cautiously (e.g., diagnostic accuracy of CMBs, very low certainty). Incorporating GRADE facilitates balanced interpretation, highlights current gaps, and underscores the need for high-quality prospective studies.

Among AIS patients, the overall pooled prevalence of CMBs was 36% when detected using SWI, which is approximately 1.7 times higher than the prevalence observed with T2* (25%). This difference was statistically significant (*p* < 0.0001) and is consistent with earlier observational studies of AIS patients, which similarly report that SWI detects between 1.2 to 1.7 times more CMBs compared to T2* [11,99]. The improved detection rate with SWI is likely attributable to its incorporation of phase imaging, which enhances magnetic susceptibility contrast, improves spatial resolution, and reduces artifacts [84]. Interestingly, when SWI and T2* were used in combination, the pooled prevalence remained at 25%. This lower-than-expected estimate likely reflects the small number of studies in this subgroup (4 studies vs. >30 studies in the individual SWI and T2* groups), which reduces representativeness and increases susceptibility to the influence of outliers. In addition, differences in T2* acquisition parameters in the combined subgroup may have attenuated the incremental benefit of SWI, effectively lowering the pooled prevalence estimate despite SWI’s known higher sensitivity. Notably, Kidwell et al. [7] reported a low CMB prevalence rate of 12% (95% CI: 0.04; 0.26), which may have disproportionately affected the combined result. Understanding these modality-dependent differences is crucial, as higher detection rates with SWI may influence clinical decision-making and risk stratification in AIS populations. The notable heterogeneity observed across studies assessing CMB prevalence likely reflects considerable methodological and clinical variability. Differences in imaging protocols, patient characteristics, CMB definitions, and interpretation differences among radiologists may further exacerbate heterogeneity across studies.

Building on these findings, the present meta-analysis has identified a range of clinical and methodological factors that influence CMB prevalence among AIS patients. A consistently higher prevalence of CMBs was observed with SWI compared to T2* across most subgroups, including those defined by age, hypertension status, geographic region, and imaging parameters. Within each imaging modality, distinct trends emerged with patient demographics. Specifically, older age and higher rates of hypertension were associated with increased CMB prevalence when using T2*. In contrast, among patients assessed with SWI, CMB prevalence appeared lower in these same subgroups. The trends observed using SWI contradict several studies [99,100,101] which consistently reported that the prevalence of CMBs rises substantially with both age and hypertension, irrespective of imaging modality. A possible explanation is that SWI, being more sensitive [11], detects a broader spectrum of CMBs even in younger or lower-risk individuals, thereby diluting the relative differences observed across age and hypertension subgroups.

Analysis of imaging parameters showed that CMB prevalence increased when FLAIR or NCCT were combined with SWI. For T2*, CMB prevalence increased with the addition of NCCT but not with FLAIR. Although previous studies have established that SWI and T2* are more sensitive than FLAIR or NCCT for detecting CMBs [11,13,69,102], few have evaluated the added diagnostic value of including FLAIR or NCCT alongside these sequences. In this meta-analysis, the pooled prevalence of CMBs detected with SWI increased by 15% with the addition of FLAIR and by 33% with NCCT. This may reflect FLAIR’s utility in distinguishing true CMBs from common mimics, such as enlarged perivascular spaces, which may influence specificity in CMB detection [16]. This meta-analysis also supports existing evidence that thinner MRI slices improve CMB detection. Our analysis found a 15% higher pooled prevalence of CMBs in studies using thin slices compared to thick slices, which is consistent with prior imaging studies, such as Nandigam et al. [15], which reported that thick-section gradient echo (GRE) detected only 33% of the CMBs identified with thin-section SWI. In addition, our analysis found that a higher magnetic field strength of 3T was correlated with increased CMB prevalence, particularly with SWI [15,103]. Conversely, the expected benefit of a higher field strength was not observed with T2*; however, this difference was not statistically significant (*p* = 0.22) and was likely influenced by outlier results from Kato et al. [19] and Wang et al. [43], who reported high CMB prevalences of 47% and 46%, respectively, when 1.5T was used. Further research is therefore necessary. These findings should be interpreted cautiously, as both the NCCT+SWI and 1.5T T2* subgroups were small, making them highly sensitive to single-study effects. Further, variations in scanner calibration, sequence optimization, and patient selection may have contributed to the observed patterns. Additional prospective studies are needed to confirm or refute these observations. Further, when stratified by continent, the highest CMB prevalence was observed in Asian populations, followed by European populations, which was evident across both modality groups. While genetic differences have been proposed [104], variations in age and comorbidities are likely major contributors, as data comparing CMB prevalence across ethnicities remain limited.

When subgrouping by stroke subtype and CMB location, the highest CMB prevalence was observed among patients with lacunar strokes and those with lobar CMBs, regardless of imaging modality. This finding aligns with the known association between lacunar strokes and small vessel disease [105], while the predominance of lobar CMBs may reflect underlying CAA, particularly in older populations [106]. Hence, this analysis builds on previous research by offering a more comprehensive evaluation of the factors linked to CMB prevalence, incorporating a wider spectrum of clinical, demographic, and imaging-related variables.

The meta-analysis also identified differences in how imaging modalities influenced the association between CMBs and prognostic outcomes. Of the studies included for prognostic outcomes, 14 underwent IVT, 5 underwent EVT, 2 had bridging therapy, and 8 had no reperfusion therapy. Overall, SWI was associated with higher odds ratios compared to T2* for sICH, HT, and poor functional outcomes, with respective odds ratios of 1.26, 1.14, and 1.10. Further, the highest odds ratios for sICH and HT outcomes were observed in the subgroups using SWI and T2*, though this subgroup was limited to only one study. While no meta-analysis directly compared SWI to T2* for CMB stroke outcomes, the association with CMBs and increased odds of sICH, HT, and 90-day poor functional outcome (mRS 3-6) is consistent with findings reported in previous studies and meta-analyses [51,107,108,109].

The diagnostic performance of CMB presence in terms of predicting clinical outcomes was modest, as reflected by the AUC values derived from the SROC analyses (Table 6). For sICH, the AUC was notably low at 0.11 when using SWI and 0.30 for T2*, indicating poor discriminatory ability of CMBs as a standalone prognostic marker. Similarly, for HT and poor 90-day functional outcomes, AUCs remained below 0.65, underscoring the limited predictive utility of CMBs and reinforcing the need for multimodal risk stratification approaches. In addition, the presence of CMBs demonstrated limited predictive value for sICH, with both SWI and T2* showing poor sensitivity (SWI: 0.05; T2: 0.09) but high specificity (SWI: 0.98; T2: 0.96). While SWI showed marginally better discriminatory ability (diagnostic odds ratio [DOR] = 3) compared to T2* (DOR = 2), both modalities exhibited overall limited predictive performance for sICH risk stratification.

For HT, SWI demonstrated higher sensitivity (0.34) than T2* (0.21) but lower specificity (0.75 vs. 0.82). DORs were low for both modalities (2 for SWI, 1 for T2*), suggesting limited predictive value. Cross-comparison data were not available for mRS scores across modalities. While SWI detects more CMBs than T2*, demonstrating greater sensitivity, the clinical prognostic value of CMB detection remains limited. This was evident from the low DORs and AUC values below 0.6 for key outcomes such as sICH, HT, and functional decline, indicating poor predictive accuracy. These findings caution against overreliance on CMB presence alone for prognostication and highlight the need for integrated, multimodal risk assessment strategies in acute stroke management.

The results align with the Living Lesion Paradigm, which reframes CMBs not as incidental remnants but as active, evolving markers of neurovascular dysfunction [8]. The higher prevalence of CMBs observed with SWI supports this perspective, suggesting that SWI captures a broader spectrum of dynamic vascular injury [110]. Further studies are warranted to assess whether SWI can detect biological processes such as angiogenesis and extracellular matrix deposition, which are known to occur during the resolution phase of other hemorrhagic pathologies [1]. This paradigm also helps explain the modest prognostic performance observed across imaging modalities, as conventional metrics may not fully capture the ongoing pathophysiological processes underlying CMBs [105,111]. In addition, CMBs appear to reflect a state of continued vulnerability rather than serving as a definitive predictor of outcome, consistent with their low sensitivity and modest DORs observed in the meta-analyses [111]. The increased odds of sICH and HT in patients with CMBs across both SWI and T2* support this paradigm by suggesting that these lesions reflect ongoing pathological processes rather than resolved hemorrhagic events. This aligns with mechanistic data indicating persistent microvascular instability in CMB regions [1]. Viewing CMBs as dynamic lesions highlights the need for sophisticated, temporally informed imaging strategies and integrated biomarkers for longitudinal monitoring and risk stratification, rather than relying on CMB detection for acute prognostic decision-making alone.

Clinically, this evolving understanding has significant implications for optimizing both CMB detection and AIS treatment strategies, particularly in managing the risks of sICH, HT, and poor functional outcomes. Identifying CMBs can prompt stricter vascular risk control and influence the use of antithrombotic therapies, especially in patients with a high CMB burden [7]. Given SWI’s improved ability to detect CMBs over T2* [11], many centers should consider adopting it if feasible. However, given that SWI is not universally available, particularly in low-resource settings, these findings must be interpreted with caution, as access disparities may limit the global applicability of SWI-based diagnostic strategies. In addition, combining SWI with complementary modalities, such as FLAIR or NCCT, can further improve specificity by distinguishing true CMBs from artifacts [16]. Despite its clinical importance, CMB detection faces challenges. Variability in MRI parameters leads to inconsistent detection, and a lack of longitudinal studies limits understanding of long-term outcomes [112]. Furthermore, current risk models for HT and sICH rarely incorporate CMB burden, and limited access to advanced MRI techniques restricts widespread use [14]. Standardized protocols, multicenter studies, and improved access are essential to enhance the clinical utility of CMB detection.

## 5. Limitations

There are several important limitations to this study that should be acknowledged. First, high levels of heterogeneity were observed across most analyses, particularly regarding CMB prevalence estimates. This variability likely reflects differences in study populations, as well as in MRI parameters, such as field strength, slice thickness, and echo time, which significantly impact CMB detection rates [112]. Standardized imaging protocols are essential to minimize inter-study heterogeneity and improve comparability. Second, varying definitions of CMBs, HT, and sICH were evident across studies, which may have introduced classification bias, influencing both prevalence and prognostic associations. These definitional discrepancies could lead to misclassification bias, either overestimating or underestimating true associations. For example, stricter sICH definitions may exclude clinically relevant hemorrhages, while broader definitions could artificially inflate prevalence and odds ratios. To ensure uniformity in research findings, establishing consistent definitions across centers is essential.

Third, several subgroup analyses, particularly those involving stroke subtypes, CMB locations, or combined SWI and T2* modalities, were based on a small number of studies. This restricts the statistical power and generalizability of these specific findings. Future research should prioritize larger studies, focusing on these subgroups, to clarify observed trends. Fourth, despite the association between CMB presence and adverse outcomes, diagnostic performance analyses revealed low sensitivity and modest DORs, indicating that CMB detection alone provides limited predictive value for sICH, HT, or poor functional outcomes. Fifth, the lack of histopathological validation poses a limitation, as all studies relied on imaging markers rather than direct tissue analysis, raising the risk of false positives from mimics [113]. Future studies should incorporate post-mortem correlation studies to clarify the true accuracy of SWI and T2* for detecting CMBs [114,115]. The identified limitations should be acknowledged when assessing the study’s results and overall implications.

## 6. Conclusions

In summary, this study found a pooled CMB prevalence of 36% with SWI and 25% with T2* among AIS patients, confirming the superior detection capability of SWI. Increased CMB prevalence was associated with older age, hypertension, lacunar stroke, lobar CMB location, thinner slice thickness, and higher field strength, though the strength and consistency of these associations varied slightly by imaging modality used. Furthermore, CMB presence was associated with adverse prognostic outcomes, including sICH, HT, and poor functional outcomes at 90 days. Although both SWI and T2* detected these associations, SWI demonstrated higher odds ratios across all outcomes, suggesting a stronger link between SWI-detected CMBs and prognosis. Despite this, the prognostic accuracy of CMB detection for predicting adverse outcomes remains limited, with low sensitivity (0.05 to 0.49) and modest DORs (2 to 3) across outcomes. To further qualify these results, our GRADE appraisal indicated that while associations with poor functional outcome are supported by moderate certainty of evidence, other findings—such as diagnostic accuracy—remain of very low certainty due to observational design, heterogeneity, and imprecision. This highlights the clinical value and the limitations of current evidence. Together, these findings underscore the utility of SWI in CMB detection and risk stratification among AIS patients, while also emphasizing the need for cautious interpretation and integration of CMB findings within a broader clinical context. Future research should standardize imaging protocols, reduce heterogeneity, and strengthen the certainty of evidence through high-quality prospective studies, ultimately supporting better identification of high-risk patients and guiding targeted management to improve outcomes.

## Figures and Tables

**Table 1:** Clinical Characteristics of Studies Included in Meta-Analysis for Acute Ischemic Stroke Patients.

Author	Year	Continent	Study Design	Cohort	Age Mean (±Standard Deviation (SD))			Male, *n* (*n*%)	Number of CMBs	CMB Definition	CMB Imaging
					Overall	Cerebral Microbleed (CMB)	No CMB				
Agbonon et al. [63]	2024	Europe	Retrospective	445	68.3 (±15.2)	71.7 (±13)	-	229 (51)	70	-	T2 Gradient Echo Imaging (T2*GRE)
Akhtar et al. [75]	2018	Asia	Retrospective	718	54.7 (±14)	-	-	594 (83)	166	<5 mm	Susceptibility Weighted Imaging (SWI)
Bai et al. [67]	2013	Asia	Prospective	113	61.6 (±10.8)	-	-	-	46	-	SWI
Bao et al. [87]	2023	Asia	Retrospective	199	-	-	-	-	92	2–10 mm	SWI
Braemswig et al. [53]	2019	Europe	Prospective	396	-	-	-	103 (26)	115	<10 mm	T2*GRE
Brauner et al. [97]	2023	Europe	Prospective	246	73.6 (±13.3)	-	-	117 (48)	72	-	T2*GRE, SWI
Brundel et al. [41]	2014	Europe	Prospective	155	-	-	-	-	19	-	T2*GRE
Capuana et al. [59]	2021	Europe	Prospective	434	68.3 (±13.3)	69 (±12.6)	68.1 (±13.8)	264 (61)	101	<10 mm	T2*GRE
Chacon-Portillo et al. [76]	2018	North America	Retrospective	292	63 (±15)	-	-	240 (82)	62	2–10 mm	SWI
Chen et al. [88]	2023	Asia	Retrospective	190	-	-	-	104 (55)	82	<10 mm	SWI
Choi et al. [54]	2019	Asia	Prospective	1532	69.4 (±11.8)	72 (±11.2)	68.9 (±11.9)	855 (56)	165	-	T2*GRE
Dannenberg et al. [42]	2014	Europe	Prospective	326	-	-	-	159 (49)	81	≤10 mm	T2*GRE
Dassan et al. [34]	2011	Europe	Retrospective	20	-	-	-	-	5	-	T2*GRE
Derraz et al. [60]	2021	Europe	Prospective	513	69.4 (±25.9)	80.8 (±15.7)	67.3 (±25.4)	243 (47)	89	≤10 mm	T2*GRE
Diker et al. [83]	2022	Europe	Retrospective	127	66.6 (±14.4)	68.5 (±12.9)	63.6 (±15.6)	74 (58)	47	<10 mm	SWI
Elnekeidy et al. [69]	2014	Africa	Prospective	46	-	-	-	-	5	-	SWI
Fan et al. [21]	2003	Asia	Prospective	121	68 (±11)	69.5 (±11)	67.1 (±10.9)	82 (68)	43	-	T2*GRE
Fiehler et al. [28]	2007	Multinational	Retrospective	570	68.3 (±13.3)	-	-	341 (60)	86	<5 mm	T2*GRE
Gao et al. [66]	2008	Asia	Retrospective	114	-	-	-	-	20	<10 mm	SWI
Gratz et al. [70]	2014	Europe	Prospective	392	68.1 (±13.7)	-	-	223 (57)	79	<5 mm	SWI
Gregoire et al. [36]	2013	Europe	Prospective	254	-	-	-	-	59	-	T2*GRE
Guo et al. [89]	2023	Asia	Retrospective	230	63.8 (±11)	66.5 (±10.8)	61.3 (±11.1)	160 (70)	111	2–10 mm	SWI
Han et al. [30]	2009	Asia	Retrospective	247	61.3 (±11.4)	64.6 (±11)	60 (±11.6)	176 (71)	72	≤5 mm	T2*GRE
Horstmann et al. [71]	2015	Europe	Prospective	645	-	-	-	-	165	≤10 mm	SWI
Hou et al. [92]	2024	Asia	Retrospective	200	68.3 (±9.5)	70.7 (±8.6)	65.3 (±10.5)	144 (72)	112	-	SWI
Huang et al. [68]	2013	Asia	Prospective	126	63.8 (±13)	64.6 (±12.7)	63.2 (±13.3)	83 (66)	63	2–10 mm	SWI
Jablonski et al. [61]	2021	Europe	Prospective	49	-	-	-	23 (47)	14	-	T2*GRE
Jeon et al. [31]	2009	Area	Retrospective	237	64 (±12.8)	-	-	142 (60)	75	≤5 mm	T2*GRE
Kakuda et al. [24]	2005	Multinational	Prospective	70	70.8 (±29.2)	70 (±32)	71 (±29)	31 (44)	11	<5 mm	T2*GRE
Kato et al. [19]	2002	Asia	Retrospective	113	-	-	-	65 (58)	53	-	T2*GRE
Khaladkar et al. [84]	2022	Asia	Prospective	20	-	-	-	-	13	-	SWI
Kidwell et al. [7]	2002	North America	Retrospective	41	-	-	-	-	5	<5 mm	T2*GRE, SWI
Ho et al. [26]	2006	Asia	Retrospective	65	-	-	-	37 (57)	25	<5 mm	T2*GRE
Kimura et al. [37]	2013	Asia	Prospective	224	76.2 (±10.6)	-	-	121 (54)	72	-	T2*GRE
Lau et al. [65]	2017	Asia	Prospective	1003	69 (±12)	-	-	601 (60)	450	<10 mm	SWI
Lau et al. [77]	2018	Asia	Prospective	1003	-	-	-	601 (60)	450	-	SWI
Lee et al. [22]	2004	Asia	Retrospective	144	64.6 (±9.1)	-	-	75 (52)	50	≤5 mm	T2*GRE
Lee et al. [62]	2022	Asia	Retrospective	577	67 (±13)	70.8 (±10.4)	66.7 (±12.8)	322 (56)	91	<10 mm	T2*GRE
Li et al. [81]	2019	Asia	Retrospective	180	71.5 (±12.4)	-	-	100 (56)	90	2–10 mm	SWI
Liang et al. [55]	2019	Asia	Prospective	563	67 (±10.2)	-	-	333 (59)	76	-	T2*GRE
Liu et al. [72]	2015	Asia	Prospective	87	67.3 (±12.5)	-	-	49 (56)	16	2–5 mm	SWI
Luo et al. [93]	2024	Asia	Retrospective	206	-	-	-	-	123	≤10 mm	SWI
Moriya et al. [38]	2013	Asia	Retrospective	71	73 (±10)	-	-	50 (70)	14	-	T2*GRE
Nagaraja et al. [85]	2021	North America	Retrospective	196	66.1 (±14)	72 (±13)	63.6 (±14.4)	98 (50)	58	2–10 mm	SWI
Nagaraja et al. [52]	2018	North America	Retrospective	366	67 (±15)	74.1 (±12.5)	64.9 (±15.2)	198 (54)	95	<10 mm	T2*GRE
Naka et al. [23]	2004	Asia	Prospective	66	-	-	-	-	12	-	T2*GRE
Naka et al. [39]	2013	Asia	Prospective	1502	72.6 (±12)	-	-	881 (59)	542	<10 mm	T2*GRE
Naka et al. [27]	2006	Asia	Prospective	183	-	-	-	-	53	-	T2*GRE
Nam et al. [56]	2019	Asia	Prospective	841	68	-	-	516 (61)	257	<10 mm	T2*GRE
Nasreldein et al. [94]	2024	Africa	Prospective	364	-	-	-	-	102	-	SWI
Nighoghossian et al. [20]	2002	Europe	Prospective	100	60 (±13)	-	-	58 (58)	20	2–5 mm	T2*GRE
Orken et al. [32]	2009	Europe	Prospective	141	65.8 (±12.2)	69.6 (±10.7)	64.7 (±12.4)	82 (58)	31	<5 mm	T2*GRE
Ozbek et al. [78]	2018	Europe	Prospective	148	68 (±14.8)	-	-	84 (57)	66	2–10 mm	SWI
Potigumjon et al. [49]	2017	Asia	Retrospective	200	61	66	60	126 (63)	39	<10 mm	T2*GRE
Purrucker et al. [79]	2018	Europe	Prospective	290	78.6	-	-	150 (52)	36	2–10 mm	SWI
Ryu et al. [58]	2020	Asia	Prospective	477	66 (±14)	-	-	294 (62)	125	≤10 mm	T2*GRE
Schlemm et al. [95]	2022	Europe	Prospective	459	68	71.7	67	289 (63)	98	≤10 mm	T2*GRE, SWI
Shahjouei et al. [50]	2017	North America	Retrospective	760	62.1 (±13.9)	-	-	391 (51)	122	≤10 mm	T2*GRE
Shi et al. [47]	2016	Asia	Prospective	206	66.8 (±17.6)	77 (±14)	65 (±18)	87 (42)	37	<10 mm	T2*GRE
Soo et al. [35]	2012	Asia	Prospective	133	67.3	67	67.4	-	23	2–10 mm	T2*GRE
Soo et al. [29]	2008	Asia	Prospective	908	68.4 (±11.9)	71.2 (±10)	67.3 (±11.8)	524 (58)	252	-	T2*GRE
Sun et al. [33]	2009	Asia	Retrospective	998	68.3 (±11.7)	71.4 (±10)	67.2 (±12)	588 (59)	273	2–10 mm	T2*GRE
Takahashi et al. [57]	2019	Asia	Prospective	69	-	-	-	45 (65)	19	-	T2*GRE
Takahashi et al. [40]	2013	Asia	Retrospective	187	74 (±11)	-	-	112 (60)	63	-	T2*GRE
Turc et al. [45]	2015	Europe	Prospective	717	-	-	-	351 (49)	150	≤10 mm	T2*GRE
Wang et al. [43]	2014	Asia	Prospective	348	65.2 (±13.1)	-	-	207 (59)	160	2–5 mm	T2*GRE
Wang et al. [90]	2023	Asia	Retrospective	581	64.3	65.6	63.5	388 (67)	225	<10 mm	SWI
Wang et al. [91]	2023	Asia	Retrospective	732	-	-	-	-	279	<10 mm	SWI
Werring et al. [25]	2005	Europe	Prospective	86	62.1 (±16.1)	-	-	57 (66)	20	<10 mm	T2*GRE
Xu et al. [82]	2021	Asia	Prospective	459	67.3 (±11.7)	69 (±11.3)	66.1 (±12)	314 (68)	187	2–10 mm	SWI
Yan et al. [73]	2015	Asia	Retrospective	333	66.2 (±13)	-	-	223 (67)	133	≤10 mm	SWI
Yan et al. [44]	2014	Asia	Prospective	121	67.3 (±12.5)	72.2 (±13)	-	77 (64)	57	≤10 mm	T2*GRE
Yang et al. [48]	2016	Asia	Prospective	348	65.2 (±13.1)	-	-	207 (59)	160	2–5 mm	T2*GRE
Zand et al. [64]	2018	North America	Retrospective	772	61.9 (±14.2)	64.9 (±13.2)	61.3 (±14.3)	398 (52)	124	≤10 mm	T2*GRE
Zand et al. [51]	2017	North America	Prospective	672	62 (±14)	64.8 (±14.1)	61 (±14)	350 (52)	103	≤10 mm	T2*GRE
Zhang et al. [86]	2022	Asia	Prospective	242	67.5 (±9.5)	69.5 (±9.9)	66.7 (±9.2)	158 (65)	71	≤10 mm	SWI
Zhang et al. [46]	2015	Asia	Retrospective	696	60	66	59	516 (74)	162	≤10 mm	T2*GRE
Zhao et al. [74]	2017	Asia	Prospective	60	62.3 (±12.5)	-	-	38 (63)	14	2–5 mm	SWI
Zhao et al. [80]	2018	Asia	Prospective	198	68.1 (±8.7)	-	-	109 (55)	91	<10 mm	SWI
Zhao et al. [96]	2022	North America	Prospective	120	59.6	-	-	65 (54)	39	<10 mm	T2*GRE, SWI

Abbreviations: CMB = cerebral microbleed, *n* = number, T2*GRE = T2 Gradient Echo Imaging, SWI = Susceptibility Weighted Imaging, SD = Standard Deviation.

**Table 2:** Rates of Clinical Risk Factors in Acute Ischemic Stroke Patients with Cerebral Microbleeds included in the Meta-Analysis.

		Clinical Risk Factors, *n* (*n*%)						
Author	Year	Atrial Fibrillation	Hyper-lipidaemia	Hypertension	Coronary Artery Disease	Prior Stroke/Transient Ischemic Stroke	Smoking	Diabetes Mellitus
Agbonon et al. [63]	2024	-	25 (14)	46 (18)	-	-	-	6 (10)
Akhtar et al. [75]	2018	-	-	-	-	-	-	-
Bai et al. [67]	2013	-	-	-	-	-	-	-
Bao et al. [87]	2023	-	-	-	-	-	-	-
Braemswig et al. [53]	2019	-	-	-	-	-	-	-
Brauner et al. [97]	2023	-	-	-	-	-	-	-
Brundel et al. [41]	2014	-	-	-	-	-	-	-
Capuana et al. [59]	2021	17 (24)	-	77 (27)	-	-	18 (22)	15 (20)
Chacon-Portillo et al. [76]	2018	-	-	-	-	-	-	-
Chen et al. [88]	2023	-	-	35 (61)	17 (47)	-	37 (44)	26 (42)
Choi et al. [54]	2019	-	-	-	-	-	-	-
Dannenberg et al. [42]	2014	-	-	-	-	-	-	-
Dassan et al. [34]	2011	-	-	-	-	-	-	-
Derraz et al. [60]	2021	38 (25)	36 (23)	65 (21)	21 (25)	19 (31)	28 (14)	14 (21)
Diker et al. [83]	2022	21 (42)	17 (38)	34 (41)	11 (44)	7 (27)	-	15 (35)
Elnekeidy et al. [69]	2014	-	-	-	-	-	-	-
Fan et al. [21]	2003	3 (50)	11 (41)	32 (38)	-	-	17 (34)	11 (28)
Fiehler et al. [28]	2007	-	-	-	-	-	-	-
Gao et al. [66]	2008	-	-	-	-	-	-	-
Gratz et al. [70]	2014	-	-	-	-	-	-	-
Gregoire et al. [36]	2013	-	-	-	-	-	-	-
Guo et al. [89]	2023	-	32 (38)	88 (53)	-	-	30 (43)	34 (45)
Han et al. [30]	2009	-	-	63 (40)	-	26 (40)	34 (26)	17 (24)
Horstmann et al. [71]	2015	-	-	-	-	-	-	-
Hou et al. [92]	2024	9 (56)	-	83 (58)	11 (61)	-	50 (55)	55 (63)
Huang et al. [68]	2013	-	14 (38)	53 (56)	-	-	18 (49)	10 (59)
Jablonski et al. [61]	2021	-	-	-	-	-	-	-
Jeon et al. [31]	2009	-	-	-	-	-	-	-
Kakuda et al. [24]	2005	-	2 (12)	8 (19)	-	-	6 (20)	4 (21)
Kato et al. [19]	2002	-	-	-	-	-	-	-
Khaladkar et al. [84]	2022	-	-	-	-	-	-	-
Kidwell et al. [7]	2002	-	-	-	-	-	-	-
Ho et al. [26]	2006	-	-	-	-	-	-	-
Kimura et al. [37]	2013	-	-	-	-	-	-	-
Lau et al. [65]	2017	-	-	-	-	-	-	-
Lau et al. [77]	2018	-	-	-	-	-	-	-
Lee et al. [22]	2004	-	-	-	-	-	-	-
Lee et al. [62]	2022	42 (15)	-	71 (20)	-	24 (24)	19 (14)	27 (17)
Li et al. [81]	2019	-	-	-	-	-	-	-
Liang et al. [55]	2019	-	-	-	-	-	-	-
Liu et al. [72]	2015	-	-	-	-	-	-	-
Luo et al. [93]	2024	-	-	-	-	-	-	-
Moriya et al. [38]	2013	-	-	-	-	-	-	-
Nagaraja et al. [85]	2021	15 (58)	27 (38)	52 (34)	15 (34)	33 (48)	-	20 (29)
Nagaraja et al. [52]	2018	14 (24)	48 (33)	67 (31)	22 (39)	25 (49)	19 (19)	23 (28)
Naka et al. [23]	2004	-	-	-	-	-	-	-
Naka et al. [39]	2013	-	-	-	-	-	-	-
Naka et al. [27]	2006	-	-	-	-	-	-	-
Nam et al. [56]	2019	-	-	-	-	-	-	-
Nasreldein et al. [94]	2024	-	-	-	-	-	-	-
Nighoghossian et al. [20]	2002	-	-	-	-	-	-	-
Orken et al. [32]	2009	-	-	27 (24)	-	7 (27)	5 (13)	6 (22)
Ozbek et al. [78]	2018	-	-	-	-	-	-	-
Potigumjon et al. [49]	2017	6 (15)	21 (18)	33 (27)	1 (10)	9 (25)	10 (20)	10 (18)
Purrucker et al. [79]	2018	-	-	-	-	-	-	-
Ryu et al. [58]	2020	-	-	-	-	-	-	-
Schlemm et al. [95]	2022	16 (32)	-	64 (26)	-	14 (24)	-	22 (30)
Shahjouei et al. [50]	2017	-	-	-	-	-	-	-
Shi et al. [47]	2016	16 (20)	10 (16)	26 (19)	11 (26)	5 (15)	-	13 (30)
Soo et al. [35]	2012	-	20 (18)	20 (20)	-	12 (22)	12 (21)	8 (20)
Soo et al. [29]	2008	19 (28)	138 (25)	200 (32)	19 (25)	83 (46)	64 (34)	76 (26)
Sun et al. [33]	2009	19 (28)	148 (25)	211 (32)	-	-	-	81 (25)
Takahashi et al. [57]	2019	-	-	-	-	-	-	-
Takahashi et al. [40]	2013	-	-	-	-	-	-	-
Turc et al. [45]	2015	-	-	-	-	-	-	-
Wang et al. [43]	2014	-	-	-	-	-	-	-
Wang et al. [90]	2023	-	82 (36)	174 (44)	-	-	-	81 (42)
Wang et al. [91]	2023	-	-	-	-	-	-	-
Werring et al. [25]	2005	-	-	-	-	-	-	-
Xu et al. [82]	2021	10 (43)	4 (31)	120 (45)	-	-	99 (44)	44 (39)
Yan et al. [73]	2015	-	-	-	-	-	-	-
Yan et al. [44]	2014	-	-	-	-	-	-	-
Yang et al. [48]	2016	-	-	-	-	-	-	-
Zand et al. [64]	2018	13 (17)	51 (20)	110 (18)	-	43 (22)	45 (16)	44 (17)
Zand et al. [51]	2017	-	-	-	-	-	-	-
Zhang et al. [86]	2022	9 (30)	-	54 (34)	16 (27)	-	26 (30)	27 (39)
Zhang et al. [46]	2015	-	124 (22)	149 (27)	-	-	68 (21)	53 (19)
Zhao et al. [74]	2017	-	-	-	-	-	-	-
Zhao et al. [80]	2018	-	-	25 (52)	-	-	44 (46)	13 (54)
Zhao et al. [96]	2022	-	-	-	-	-	-	-

Abbreviations: CMB = cerebral microbleed, *n* = number., AF = atrial fibrillation, HL = hyperlipidaemia, HTN = hypertension, CAD = coronary artery disease, PS = prior stroke, TIA = transient ischemic attack, DM = diabetes mellitus.

**Table 3:** Prognostic Outcomes of Studies Selected for Meta-Analysis.

Author	Year	Reperfusion Therapy	Symptomatic Intracranial Hemorrhage (sICH) Definition	sICH, *n* (*n*%)			Hemorrhagic Transformation (HT), *n* (*n*%)			Modified Ranking Scale (mRS) 3–6 at 90 Days, *n* (*n*%)		
				Overall	Cerebral Microbleed (CMB)	No-CMB	Overall	CMB	No-CMB	Overall	CMB	No-CMB
Agbonon et al. [63]	2024	Endovascular Thrombolysis (EVT)	ECASS-II	34 (7.6)	6 (1.4)	28 (6.3)	-	-	-	194 (43.6)	31 (7.0)	163 (36.6)
Capuana et al. [59]	2021	Intravenous Thrombolysis (IVT)	SITS-MOST	13 (3.0)	7 (1.6)	6 (1.4)	-	-	-	130 (30.0)	39 (9.0)	91 (21.0)
Chacon-Portillo et al. [76]	2018	IVT	NINDS	6 (2.0)	3 (1.0)	3 (1.0)	46 (15.8)	12 (4.1)	34 (11.6)	63 (21.6)	16 (6.2)	42 (14.4)
Choi et al. [54]	2019	IVT/EVT	ECASS-I	69 (4.5)	17 (1.1)	52 (3.4)	420 (27.4)	66 (4.3)	354 (23.1)	865 (56.4)	103 (6.7)	763 (49.8)
Dannenberg et al. [42]	2014	IVT	ECASS-III	10 (3.1)	7 (2.1)	3 (0.9)	-	-	-	158 (48.4)	50 (15.3)	108 (33.1)
Derraz et al. [60]	2021	EVT	ECASS-II	66 (12.9)	15 (2.9)	51 (9.9)	-	-	-	281 (54.8)	59 (11.5)	222 (43.3)
Elnekeidy et al. [69]	2014	-	-	-	-	-	10 (21.7)	1 (2.2)	9 (19.6)	-	-	-
Fiehler et al. [28]	2007	IVT	ECASS-I	18 (3.2)	5 (0.9)	13 (2.3)	-	-	-	-	-	-
Gratz et al. [70]	2014	IVT/EVT	PROACT-II	21 (5.4)	3 (0.8)	18 (4.6)	-	-	-	193 (49.2)	52 (13.3)	141 (36.0)
Kakuda et al. [24]	2005	IVT	ECASS-II	7 (10.0)	0 (0)	7 (10.0)	32 (45.7)	3 (4.3)	29 (41.4)	-	-	-
Khaladkar et al. [84]	2022	-	-	-	-	-	18 (90)	13 (65)	5 (25)	-	-	-
Kidwell et al. [7]	2002	IVT	-	-	-	-	15 (36.6)	2 (4.9)	13 (31.7)	-	-	-
Ho et al. [26]	2006	IVT	-	5 (12.2)	3 (7.3)	2 (4.9)	17 (41.5)	8 (19.5)	9 (22.0)	-	-	-
Lee et al. [62]	2022	EVT	-	-	-	-	170 (29.5)	32 (55.5)	138 (21.9)	288 (49.9)	59 (10.2)	229 (39.7)
Liu et al. [72]	2015	-	-	-	-	-	17 (19.5)	5 (5.7)	12 (13.8)	-	-	-
Moriya et al. [38]	2013	IVT	-	-	-	-	26 (36.6)	6 (8.5)	20 (28.2)	-	-	-
Nagaraja et al. [52]	2018	-	-	-	-	-	87 (23.8)	32 (8.7)	55 (15.0)	-	-	-
Nagaraja et al. [85]	2021	-	-	-	-	-	22 (11.2)	6 (3.1)	16 (8.2)	36 (18.4)	12 (6.1)	24 (12.2)
Nighoghossian et al. [20]	2002	IVT	-	-	-	-	26 (26.0)	10 (10.0)	16 (16.0)	-	-	-
Ozbek et al. [78]	2018	-	-	-	-	-	41 (27.7)	18 (12.2)	23 (15.5)	-	-	-
Schlemm et al. [95]	2022	IVT	SITS-MOST, ECASS-II, ECASS-III, NINDS	26 (5.7)	11 (2.4)	15 (3.3)	102 (22.2)	21 (4.6)	46 (10.0)	125 (27.2)	34 (7.4)	91 (19.8)
Shi et al. [47]	2016	EVT	-	-	-	-	91 (44.2)	14 (6.8)	77 (37.4)	-	-	-
Soo et al. [35]	2012	EVT	-	-	-	-	7 (5.3)	1 (0.8)	6 (4.5)	-	-	-
Takahashi et al. [40]	2013	-	-	-	-	-	27 (14.4)	5 (2.7)	22 (11.8)	-	-	-
Yan et al. [73]	2015	IVT	ECASS-II	8 (2.4)	6 (1.8)	2 (0.6)	102 (30.6)	48 (14.4)	54 (16.2)	206 (61.9)	140 (42.0)	66 (19.8)
Yang et al. [48]	2016	-	-	-	-	-	35 (10.0)	10 (2.9)	25 (7.2)	-	-	-
Zand et al. [51]	2017	IVT	ECASS-II	25 (3.7)	5 (0.7)	20 (3.0)	-	-	-	-	-	-
Zand et al. [64]	2018	IVT	-	-	-	-	6 (0.8)	3 (0.4)	3 (0.4)	-	-	-
Zhao et al. [74]	2017	IVT	ECASS-II	2 (3.3)	2 (3.3)	0 (0)	-	-	-	-	-	-

Abbreviations: CMB = cerebral microbleed, *n* = number, IVT = intravenous thrombolysis, EVT = endovascular thrombolysis, sICH = symptomatic intracranial hemorrhage, HT = hemorrhagic transformation, mRS = Modified Rankin Scale.

**Table 4:** Meta-Analysis Results for Prevalence of Cerebral Microbleeds: Summary Effects and Heterogeneity.

Modality	Subgroup	Pooled Prevalence (Effect Size)	95% Confidence Interval	Weight (%)	Heterogeneity χ^2^ (Degrees of Freedom)	*p*-Value	I^2^ (%)	z-Score	*p*-Value (z-Test)
T2 Gradient Echo Imaging (T2*)	-	0.25	0.22–0.28	57.74	844.41 (45)	0	94.67	28.82	0
Susceptibility Weighted Imaging (SWI)	-	0.36	0.31–0.41	37.44	563.55 (29)	0	94.85	25.61	0
Both	-	0.25	0.18–0.32	4.82	12.99 (3)	0	76.90	11.67	0
Overall	-	0.29	0.26–0.31	100	1912.84 (79)	0	95.87	35.04	0
Age									
T2*	<65 years	0.22	0.18–0.26	29.20	75.77 (9)	0	88.12	18.58	0
	≥65 years	0.25	0.21–0.30	70.80	674.41 (23)	0	96.59	19.14	0
	Overall	0.24	0.21–0.28	100	779.72 (33)	0	95.77	24.20	0
SWI	<65 years	0.36	0.27–0.47	31.66	103.61 (5)	0	95.17	11.57	0
	≥65 years	0.35	0.28–0.43	68.34	261.71 (12)	0	95.41	15.56	0
	Overall	0.36	0.30–0.42	100	377.01 (18)	0	95.23	19.92	0
Hypertension									
T2*	<65% HTN	0.21	0.17–0.27	44.34	161.27 (11)	0	93.18	14.73	0
	≥65% HTN	0.26	0.23–0.29	55.66	107.71 (14)	0	87.00	27.15	0
	Overall	0.24	0.21–0.27	100	300.82 (26)	0	91.38	27.94	0
SWI	<65% HTN	0.37	0.27–0.48	26.22	65.62 (4)	0	93.90	10.96	0
	≥65% HTN	0.36	0.29–0.43	73.78	351.70 (13)	0	96.30	16.52	0
	Overall	0.36	0.30–0.42	100	418.37 (18)	0	95.70	20.24	0
Fluid Attenuated Inversion Recovery (FLAIR)									
T2*	FLAIR	0.24	0.21–0.27	60.69	333.22 (27)	0	91.90	25.27	0
	No FLAIR	0.26	0.20–0.31	39.31	499.28 (17)	0	96.60	15.95	0
	Overall	0.25	0.22–0.28	100	844.41 (45)	0	94.67	28.82	0
SWI	FLAIR	0.38	0.31–0.44	63.04	348.57 (16)	0	95.41	18.86	0
	No FLAIR	0.33	0.25–0.42	36.96	185.34 (9)	0	95.14	12.30	0
	Overall	0.36	0.31–0.41	100	555.50 (26)	0	95.32	22.70	0
Non-contrast Computed Tomography (NCCT)									
T2*	NCCT	0.27	0.21–0.33	35.74	573.23 (15)	0	97.38	14.89	0
	No NCCT	0.24	0.21–0.26	64.26	260.83 (29)	0	88.88	28.43	0
	Overall	0.25	0.22–0.28	100	844.41 (45)	0	94.67	28.82	0
SWI	NCCT	0.44	0.34–0.54	22.39	62.59 (5)	0	92.01	13.27	0
	No NCCT	0.33	0.28–0.39	77.61	467.38 (20)	0	95.72	19.05	0
	Overall	0.36	0.31–0.41	100	555.50 (26)	0	95.32	22.70	0
Field Strength in Tesla (T)									
T2*	1.5	0.27	0.23–0.31	68.57	252.74 (21)	0	91.76	22.77	0
	3T	0.23	0.18–0.28	31.43	112.65 (8)	0	92.90	16.68	0
	Overall	0.25	0.22–0.29	100	460.20 (30)	0	93.48	26.04	0
SWI	1.5T	0.36	0.26–0.47	35.48	106.63 (7)	0	93.44	10.85	0
	3T	0.37	0.31–0.43	64.52	261.40 (13)	0	95.03	19.39	0
	Overall	0.37	0.32–0.42	100	370.43 (21)	0	94.33	23.04	0
Slice Thickness									
Overall	Thin ≤ 2 mm	0.40	0.32–0.49	13.36	139.05 (10)	0	92.81	14.10	0
	Medium 2.1–4.9 mm	0.23	0.18–0.28	5	10.84 (3)	0.01	72.33	15.56	0
	Thick ≥ 5 mm	0.25	0.22–0.29	41.78	545.62 (32)	0	94.14	25.02	0
	Overall	0.28	0.25–0.31	100	809.02 (47)	0	94.19	29.72	0
Region									
T2*	Asia	0.28	0.24–0.33	59.14	645.90 (26)	0	95.97	21.40	0
	Europe	0.21	0.19–0.24	27.25	41.79 (12)	0	71.29	25.63	0
	North America	0.18	0.14–0.22	9.4	19.97 (3)	0	84.97	16.17	0
	Multinational	0.15	0.12–0.18	4.21	-	-	-	18.06	0
	Overall	0.25	0.22–0.28	100	844.41 (45)	0	94.67	28.82	0
SWI	Africa	0.26	0.22–0.30	6.4	-	-	-	19.45	0
	Asia	0.41	0.37–0.46	68.91	260.13 (19)	0	92.70	28.14	0
	Europe	0.27	0.18–0.37	17.63	69.37 (4)	0	94.23	9.34	0
	North America	0.24	0.21–0.28	7.06	-	-	-	21.50	0
	Overall	0.36	0.32–0.41	100	559.19 (28)	0	94.99	25.44	0
Stroke Subtype									
T2*	Atherothrombotic	0.25	0.12–0.39	28.03	46.29 (4)	0	91.36	5.74	0
	Lacunar	0.39	0.25–0.53	29.73	35.05 (4)	0	88.59	8.24	0
	Cardioembolic	0.24	0.14–0.35	6.59	11.31 (4)	0.02	64.65	7.09	0
	Undetermined	0.27	0.20–0.33	17.11	-	-	-	12.71	0
	Overall	0.29	0.23–0.36	100	119.90 (17)	0	85.82	14.31	0
SWI	Atherothrombotic	0.23	0.08–0.42	27.31	104.67 (4)	0	96,18	4.19	0
	Lacunar	0.26	0.17–0.37	26.62	19.57 (4)	0	79.56	8.45	0
	Cardioembolic	0.25	0.11–0.43	26.37	61.15 (4)	0	93.46	4.96	0
	Undetermined	0.20	0.10–0.32	19.7	11.40 (3)	0.01	73.69	5.60	0
	Overall	0.24	0.18–0.30	100	229.98 (18)	0	92.17	11.94	0
Cerebral Microbleed Location									
T2*	Deep	0.33	0.20–0.47	19.76	60.02 (6)	0	90.00	7.47	0
	Infratentorial	0.08	0.02–0.19	13.69	19.12 (4)	0	79.08	3.15	0
	Lobar	0.37	0.29–0.46	34.78	93.38 (11)	0	88.22	13.21	0
	Mixed	0.46	0.36–0.55	31.76	84.44 (10)	0	88.16	14.15	0
	Overall	0.34	0.28–0.41	100	446 (34)	0	92.38	16.16	0
SWI	Deep	0.18	0.14–0.21	23.04	19.94 (8)	0.01	59.87	16.52	0
	Infratentorial	0.12	0.07–0.19	23.04	86.75 (8)	0	90.78	6.55	0
	Lobar	0.29	0.24–0.34	28.25	45.14 (10)	0	77.85	18.79	0
	Mixed	0.49	0.39–0.60	25.68	155.02 (9)	0	94.19	13.46	0
	Overall	0.27	0.21–0.33	100	1021.49 (38)	0	96.28	14.86	0

Abbreviations: T2* = T2 Gradient Echo Imaging, SWI = Susceptibility Weighted Imaging, FLAIR = fluid attenuated inversion recovery, NCCT = non-contrast computed tomography, T = tesla.

**Table 5:** Meta-Analysis Results for Association of Cerebral Microbleeds with Prognostic Outcomes: Summary Effects and Heterogeneity.

Outcome	Modality	Effect Measure	Summary Effects	Heterogeneity *⍺*		Heterogeneity Variance Estimates			
			DerSimonian and Laird Random-Effects Method (REDL)	Tests of Overall Effect	Cochran’s Q	H	I^2^ ≤ *	*p*-Value	τ^2^ ≤ ^†^
			Odds Ratio (OR) (95% Confidence Interval)						
Symptomatic intracranial hemorrhage (sICH)	T2 Gradient Echo Imaging (T2*)	OR	2.13 [1.435; 3.160]	*p* = 0.000, z = 3.754	11.08	1.18	27.8%	0.197	0.0949
	Susceptibility Weighted Imaging (SWI)	OR	2.687 [0.722; 10.007]	*p* = 0.141, z = 1.474	6.86	1.51	56.3%	0.076	0.972
	Both	OR	2.916 [1.294; 6.574]	*p* = 0.010, z = 2.581	0.00	-	-	-	0
	Overall	OR	2.216 [1.555; 3.159]	*p* = 0.000, z = 4.402	18.49	1.19	29.7%	0.140	0.122
Hemorrhagic transformation (HT)	T2*	OR	1.229 [0.820; 1.843]	*p* = 0.319, z = 0.997	32.95	1.73	66.6%	0.001	0.282
	SWI	OR	1.402 [0.910; 2.163]	*p* = 0.125, z = 1.535	8.64	1.20	30.6%	0.195	0.0956
	Both	OR	1.788 [1.033; 3.094]	*p* = 0.038, z = 2.076	0.70	0.84	0.0%	0.401	0
	Overall	OR	1.332 [1.013; 1.750]	*p* = 0.040, z = 2.054	1.16	1.47	53.5%	0.002	0.174
Modified Ranking Scale (mRS) 3–6 at 90 Days	T2*	OR	1.572 [1.282; 1.927]	*p* = 0.000, z = 4.346	6.06	1.10	17.5%	0.300	0.0114
	SWI	OR	1.727 [1.303; 2.289]	*p* = 0.000, z = 3.798	2.68	0.95	0.0%	0.444	0
	Both	OR	1.579 [0.976; 2.555]	*p* = 0.063, z = 1.859	0.00	-	-	-	0
	Overall	OR	1.606 [1.387; 1.858]	*p* = 0.000, z = 6.344	9.09	0.95	0.0%	0.524	0

Abbreviations: T2* = T2 Gradient Echo Imaging, SWI = Susceptibility Weighted Imaging, sICH = symptomatic intracranial hemorrhage, HT = hemorrhagic transformation, mRS = Modified Rankin Scale, OR = odds ratio, CI = confidence interval, REDL = DerSimonian and Laird random-effects method, Q = heterogeneity measures were calculated from data with 95% confidence intervals (95% CI), based on non-central χ^2^ (common effect) distribution for Cochran’s Q test, H = relative excess in Cochran’s Q over degrees of freedom, I^2^ = proportion of total variation in effect estimate due to between study heterogeneity (based on Cochran’s Q test), τ^2^ = between-study variance to test comparisons of heterogeneity among subgroups, * = values of I^2^ ≤ are percentages, *α* = heterogeneity measures were calculated from the data with 95% Cis based on gamma (random effects) distribution for Q, † = heterogeneity variance estimates (tau≤) were derived from the DerSimonian and Laird method.

**Table 6:** Summary of Diagnostic Performance and Heterogeneity from MIDAS Meta-Analysis.

Outcome	Modality	Parameter	Estimate	95% Confidence Interval (CI)
Symptomatic Intracranial Hemorrhage (sICH)	Susceptibility Weighted Imaging (SWI)	Sensitivity	0.05	[0.03; 0.08]
		Specificity	0.98	[0.95; 0.99]
		Positive Likelihood Ratio	2.8	[0.7; 11.2
		Negative Likelihood Ratio	0.97	[0.93; 0.1.01]
		Diagnostic Odds Ratio	3	[1; 12]
		Pretest Probability of Disease	0.04	-
		Area under ROC Curve (AUROC)	0.11	[0.08; 0.14]
		Interstudy Variation in Sensitivity (ICC_SEN)	0.01	[0.00; 0.07]
		Interstudy Variation in Specificity (ICC_SPE)	0.17	[0.00; 0.50]
		Heterogeneity (Chi-square)	2.333, degrees of freedom (df) = 2, *p* = 0.156	
		Inconsistency (I^2^)	14	[0; 100]
	T2 Gradient Echo Imaging (T2*)	Sensitivity	0.09	[0.07; 0.12]
		Specificity	0.96	[0.93; 0.97]
		Positive Likelihood Ratio	2.1	[1.4; 3.1]
		Negative Likelihood Ratio	0.95	[0.93; 0.97]
		Diagnostic Odds Ratio	2	[1; 3]
		Pretest Probability of Disease	0.16	-
		AUROC	0.30	[ 0.26; 0.34]
		ICC_SEN	0.02	[0.00; 0.07]
		ICC_SPE	0.11	[0.00; 0.22]
		Heterogeneity (Chi-square)	29.382, df = 2, *p* < 0.0001	
		I^2^	93	[87; 99]
Hemorrhagic Transformation (HT)	SWI	Sensitivity	0.34	[0.15; 0.61]
		Specificity	0.75	[0.62; 0.85]
		Positive Likelihood Ratio	1.4	[1.0; 2.0]
		Negative Likelihood Ratio	0.87	[0.69, 1.11]
		Diagnostic Odds Ratio	2	[1, 3]
		Pretest Probability of Disease	0.23	-
		AUROC	0.65	[0.61; 0.69]
		ICC_SEN	0.37	[0.03; 0.72]
		ICC_SPE	0.16	[0.00; 0.37]
		Heterogeneity (Chi-square)	44.168, df = 2, *p* < 0.001	-
		I^2^	95	[92; 99]
	T2*	Sensitivity	0.21	[0.12; 0.35]
		Specificity	0.82	[0.69; 0.90]
		Positive Likelihood Ratio	1.2	[0.8; 1.7]
		Negative Likelihood Ratio	0.96	[0.88; 1.05]
		Diagnostic Odds Ratio	1	[1; 2]
		Pretest Probability of Disease	0.21	-
		AUROC	0.52	[0.48; 0.56]
		ICC_SEN	0.30	[0.10; 0.50]
		ICC_SPE	0.32	[0.13; 0.52]
		Heterogeneity (Chi-square)	334.234, df = 2, *p* < 0.001	-
		I^2^	99	[99; 100]
Modified Rankin Scale (mRS) 3-6 at 90 days	Overall	Sensitivity	0.49	[0.41; 0.58]
		Specificity	0.62	[0.54; 0.69]
		Positive Likelihood Ratio	1.3	[1.2; 1.4]
		Negative Likelihood Ratio	0.82	[0.75; 0.89]
		Diagnostic Odds Ratio	2	[1; 2]
		Pretest Probability of Disease	0.46	-
		AUROC	0.58	[0.54; 0.62]
		ICC_SEN	0.09	[0.05; −0.12]
		ICC_SPE	0.08	[0.05; 0.10]
		Heterogeneity (Chi-square)	170.018, df = 2, *p* < 0.0001	-
		I^2^	99	[98; 99]

Abbreviations: sICH = symptomatic intracranial hemorrhage, HT = hemorrhagic transformation, mRS = Modified Rankin Scale, CI = confidence interval, AUROC = area under ROC curve, ICC_SEN = interstudy variation in sensitivity, ICC_SPE = interstudy variation in specificity, I^2^ = inconsistency, df = degrees of freedom, *p* = *p*-value, T2* = T2 Gradient Echo Imaging, SWI = Susceptibility Weighted Imaging.

**Table 7:** GRADE Summary of Findings: Cerebral Microbleeds (CMBs) in Acute Ischemic Stroke (AIS)—SPOT-CMB Study.

Outcome	No. of Studies (Participants)	Study Design	Relative Effect (95% CI)	Assumed Risk (control)	Risk with CMBs	Absolute Effect	Certainty of Evidence	Reasons
Symptomatic intracerebral hemorrhage (sICH)	14 (~6163)	Observational (meta-analysis, random-effects)	OR 2.22 (1.56–3.16)	40 per 1000	88 per 1000	48 more per 1000	⊕⊕◯◯ Low to Moderate	−1 risk of bias (variable definitions), −1 imprecision (subgroup variability), +1 consistent association
Hemorrhagic transformation (HT)	21 (~6049)	Observational (meta-analysis, random-effects)	OR 1.33 (1.01–1.75)	150 per 1000	190 per 1000	40 more per 1000	⊕⊕◯◯ Low	−1 risk of bias, −1 inconsistency (I^2^ = 53.5%), −1 indirectness (definitions variable)
Poor functional outcome (mRS 3–6 at 90 days)	11 (~5499)	Observational (meta-analysis, random-effects)	OR 1.61 (1.39–1.86)	350 per 1000	470 per 1000	120 more per 1000	⊕⊕⊕◯ Moderate	−1 risk of bias, +1 consistency (I^2^ = 0%)
CMB prevalence by imaging modality (SWI vs. T2*)	80 (~28,383)	Observational (meta-analysis)	SWI 36% (95% CI: 31–41); T2* 25% (22–28)	—	—	11% higher detection with SWI	⊕⊕◯◯ Low	−1 inconsistency (high heterogeneity), −1 indirectness, +1 strong magnitude of effect
Diagnostic accuracy for sICH prediction	14 (~6163)	Observational (diagnostic meta-analysis)	AUC 0.29; DOR 2–3	—	—	Poor sensitivity (<10%) but high specificity (>95%)	⊕◯◯◯ Very low	−1 risk of bias, −1 indirectness, −1 imprecision

GRADE Working Group grades of evidence. ⊕⊕⊕⊕ High: Very confident that the true effect lies close to the estimate. ⊕⊕⊕◯ Moderate: Moderately confident; true effect likely close but may differ. ⊕⊕◯◯ Low: Limited confidence; true effect may differ substantially. ⊕◯◯◯ Very low: Very little confidence; true effect likely substantially different. Abbreviations: AIS = acute ischemic stroke; CMBs = cerebral microbleeds; SPOT-CMB = Susceptibility-weighted imaging and Prognostic Outcomes in Acute Stroke—Cerebral Microbleeds study; OR = odds ratio; DOR = diagnostic odds ratio; AUC = area under the ROC curve; SWI = susceptibility-weighted imaging; T2* = T2*-weighted imaging.

## Data Availability

The original contributions presented in this study are included in the article. Further inquiries can be directed to the corresponding author.

## Supplementary Materials

The following supporting information can be downloaded at: https://www.mdpi.com/article/10.3390/medicina61091566/s1, Search strategy; Supplemental Table S1: PRISMA-2020 Checklist; Supplemental Table S2: MOOSE Checklist; Supplemental Table S3: Modified Jadad Analysis for Methodological Quality; Supplemental Table S4: Funding Bias Scores for Studies; Supplemental Table S5: Outputs from Egger’s Test for Publication Bias for Association Variables; Supplemental Table S6: Outputs from Deeks’ Test for Small Study Effects and Publication Bias for Association Variables; Supplemental Figure S1: Forest Plots of CMB Prevalence, Stratified by Age, Hypertension and Regional Variation; Supplemental Figure S2: Forest Plots CMB Prevalence, Stratified by Use of FLAIR, NCCT and Slice Thickness; Supplemental Figure S3: Forest Plots of CMB Prevalence, Stratified by Field Strength, Stroke Subtype and CMB Location; Supplemental Figure S4: Graphs of Egger’s Regression Test for Meta-Analysis on the Association between CMBs and Prognostic Outcomes; Supplemental Figure S5: Graphs of Funnel Plot for Meta-Analysis on the Association between CMBs and Prognostic Outcomes; Supplemental Figure S6: Sensitivity Analysis on Association between CMBs and Prognostic Outcomes; Supplemental Figure S7: Graphs of ROC Plot for Meta-Analysis on the Association between CMBs and Prognostic Outcomes; Supplemental Figure S8: Graphs of Deeks’ Funnel Plot for Meta-Analysis on the Association between CMBs and Prognostic Outcomes; Supplemental Figure S9: Graphs on Fagan’s Plot for Meta-Analysis on the Association between CMBs and Prognostic Outcome.
