# Supplementary material for: Comparative Diagnostic and Prognostic Performance of SWI and T2-Weighted MRI in Cerebral Microbleed Detection Following Acute Ischemic Stroke: A Meta-Analysis and SPOT-CMB Study
Source: Medicina (Kaunas). 2025 Aug 30;61(9):1566. doi: 10.3390/medicina61091566 (PMC12471313; doi:10.3390/medicina61091566)
Supplement: Supplementary file 1 [file medicina-61-01566-s001.zip › medicina-3813276-supplementary.pdf]

Supplemental Information

List of Contents

1. **Search Strategy (Keywords/MeSH Terms)**
    a. PubMed Search Strategy
    b. Embase Search Strategy
    c. Cochrane Library Search Strategy
    d. Other Sources
2. **Supplemental Tables**
    a. **Supplemental Table 1**: PRISMA-2020 Checklist
    b. **Supplemental Table 2**: MOOSE Checklist
    c. **Supplemental Table 3**: Modified Jadad Analysis for Methodological Quality
    d. **Supplemental Table 4**: Funding Bias Scores for Studies
    e. **Supplemental Table 5**: Outputs from Egger's Test for Publication Bias for Association Variables
    f. **Supplemental Table 6**: Outputs from Deeks' Test for Small Study Effects and Publication Bias for Association Variables
3. **Supplemental Figures**
    a. **Supplemental Figure 1**: Forest Plots of CMB Prevalence, Stratified by Age, Hypertension and Regional Variation
    b. **Supplemental Figure 2**: Forest Plots of CMB Prevalence, Stratified by Use of FLAIR, NCCT and Slice Thickness
    c. **Supplemental Figure 3**: Forest Plots of CMB Prevalence, Stratified by Field Strength, Stroke Subtype and CMB Location
    d. **Supplemental Figure 4**: Graphs of Egger's Regression Test for Meta-Analysis on the Association between CMBs and Prognostic Outcomes
    e. **Supplemental Figure 5**: Graphs of Funnel Plot for Meta-Analysis on the Association between CMBs and Prognostic Outcomes
    f. **Supplemental Figure 6**: Sensitivity Analysis on Association between CMBs and Prognostic Outcomes
    g. **Supplemental Figure 7**: Graphs of ROC Plot for Meta-Analysis on the Association between CMBs and Prognostic Outcomes
    h. **Supplemental Figure 8**: Graphs of Deeks' Funnel Plot for Meta-Analysis on the Association between CMBs and Prognostic Outcomes
    i. **Supplemental Figure 9**: Graphs of Fagan's Plot for Meta-Analysis on the Association between CMBs and Prognostic Outcomes

1. Search Strategy (Keywords/MeSH Terms)

a. PubMed Search Strategy

Search Query: ("Susceptibility-Weighted Imaging"[Mesh] OR "SWI"[tiab] OR "Susceptibility Weighted Imaging"[tiab] OR "FLAIR"[tiab] OR "Fluid-Attenuated Inversion Recovery"[tiab] OR "Fluid Attenuated Inversion Recovery"[tiab] OR "T2 Star"[tiab] OR "T2*"[tiab] OR "T2-star imaging"[tiab] OR "T2 Star Weighted Imaging"[tiab] OR "Computed Tomography"[Mesh] OR "Non-Contrast Computed Tomography"[tiab] OR "NCCT"[tiab] OR "Noncontrast CT"[tiab] OR "Non-contrast CT"[tiab]) AND ("Cerebral Microbleeds"[Mesh] OR "CMB"[tiab] OR "Cerebral Microhemorrhage"[tiab] OR "Cerebral Microhemorrhages"[tiab] OR "Microbleed"[tiab] OR "Microbleeds"[tiab] OR "Lobar Microbleeds"[tiab] OR "Deep Microbleeds"[tiab] OR "Cortical Microbleeds"[tiab] OR "Subcortical Microbleeds"[tiab] OR "Periventricular Microbleeds"[tiab]) AND ("Ischemic Stroke"[Mesh] OR "Acute Ischemic Stroke"[tiab] OR "AIS"[tiab] OR "Cerebral Ischemia"[Mesh] OR "Brain Ischemia"[Mesh] OR "Cerebral Infarction"[Mesh] OR "Acute Stroke"[tiab] OR "Acute Cerebral Infarction"[tiab])

Filters applied: Humans, English, Adult: 19+ years, from 2000-2025.
Results: 212

b. Embase Search Strategy

Search Query: ("Susceptibility-Weighted Imaging" OR "SWI" OR "Susceptibility Weighted Imaging" OR "FLAIR" OR "Fluid-Attenuated Inversion Recovery" OR "Fluid Attenuated Inversion Recovery" OR "T2" OR "T2-star imaging" OR "T2 Star Weighted Imaging" OR "Computed Tomography" OR "Non-Contrast Computed Tomography" OR "NCCT" OR "Noncontrast CT" OR "Non-contrast CT") AND ("Cerebral Microbleeds" OR "CMB" OR "Cerebral Microhemorrhage" OR "Cerebral Microhemorrhages" OR "Microbleed" OR "Microbleeds" OR "Lobar Microbleeds" OR "Deep Microbleeds" OR "Cortical Microbleeds" OR "Subcortical Microbleeds" OR "Periventricular Microbleeds") AND ("Ischemic Stroke" OR "Acute Ischemic Stroke" OR "AIS" OR "Cerebral Ischemia" OR "Brain Ischemia" OR "Cerebral Infarction" OR "Acute Stroke" OR "Acute Cerebral Infarction")

Filters applied: Human, English Language, Adult: 18+ years, from 2000-2025.
Results: 490

c. Cochrane Library Search Strategy

Search Query: ("Susceptibility-Weighted Imaging" OR "SWI" OR "Susceptibility Weighted Imaging" OR "FLAIR" OR "Fluid-Attenuated Inversion Recovery" OR "Fluid Attenuated Inversion Recovery" OR "T2" OR "T2-star imaging" OR "T2 Star Weighted Imaging" OR "Computed Tomography" OR "Non-Contrast Computed Tomography" OR "NCCT" OR "Noncontrast CT" OR "Non-contrast CT") AND ("Cerebral Microbleeds" OR "CMB" OR "Cerebral Microhemorrhage" OR "Cerebral Microhemorrhages" OR "Microbleed" OR "Microbleeds" OR "Lobar Microbleeds" OR "Deep Microbleeds" OR "Cortical Microbleeds" OR "Subcortical Microbleeds" OR "Periventricular Microbleeds") AND ("Ischemic Stroke" OR "Acute Ischemic Stroke" OR "AIS" OR "Cerebral Ischemia" OR "Brain Ischemia" OR "Cerebral Infarction" OR "Acute Stroke" OR "Acute Cerebral Infarction")

Filters applied: English, from 2000-2025.
Results: 34

d. Scopus

Search Query: ("Susceptibility-Weighted Imaging" OR "SWI" OR "Susceptibility Weighted Imaging" OR "FLAIR" OR "Fluid-Attenuated Inversion Recovery" OR "Fluid Attenuated Inversion Recovery" OR "T2" OR "T2-star imaging" OR "T2 Star Weighted Imaging" OR "Computed Tomography" OR "Non-Contrast Computed Tomography" OR "NCCT" OR "Noncontrast CT" OR "Non-contrast CT") AND ("Cerebral Microbleeds" OR "CMB" OR "Cerebral Microhemorrhage" OR "Cerebral Microhemorrhages" OR "Microbleed" OR "Microbleeds" OR "Lobar Microbleeds" OR "Deep Microbleeds" OR "Cortical Microbleeds" OR "Subcortical Microbleeds" OR "Periventricular Microbleeds") AND ("Ischemic Stroke" OR "Acute Ischemic Stroke" OR "AIS" OR "Cerebral Ischemia" OR "Brain Ischemia" OR "Cerebral Infarction" OR "Acute Stroke" OR "Acute Cerebral Infarction")

Filters applied: Human, English, from 2000-2025
Results: 378

e. Web of Science

Search Query: ("Susceptibility-Weighted Imaging" OR "SWI" OR "Susceptibility Weighted Imaging" OR "FLAIR" OR "Fluid-Attenuated Inversion Recovery" OR "Fluid Attenuated Inversion Recovery" OR "T2" OR "T2-star imaging" OR "T2 Star Weighted Imaging" OR "Computed Tomography" OR "Non-Contrast Computed Tomography" OR "NCCT" OR "Noncontrast CT" OR "Non-contrast CT") AND ("Cerebral Microbleeds" OR "CMB" OR "Cerebral Microhemorrhage" OR "Cerebral Microhemorrhages" OR "Microbleed" OR "Microbleeds" OR "Lobar Microbleeds" OR "Deep Microbleeds" OR "Cortical Microbleeds" OR "Subcortical Microbleeds" OR "Periventricular Microbleeds") AND ("Ischemic Stroke" OR "Acute Ischemic Stroke" OR "AIS" OR "Cerebral Ischemia" OR "Brain Ischemia" OR "Cerebral Infarction" OR "Acute Stroke" OR "Acute Cerebral Infarction")

Filters applied: English, from 2000-2025
Results: 340

f. Other Sources

Additional sources identified through Google Scholar
Results:

2. Supplemental Tables

a. Supplemental Table 1: PRISMA-2020 Checklist

3. Section and Topic	Item #	Checklist item	Location where item is reported
**TITLE**			
Title	1	Identify the report as a systematic review.	1
**ABSTRACT**			
Abstract	2	See the PRISMA 2020 for Abstracts checklist.	2
**INTRODUCTION**			
Rationale	3	Describe the rationale for the review in the context of existing knowledge.	3
Objectives	4	Provide an explicit statement of the objective(s) or question(s) the review addresses.	3
**METHODS**			
Eligibility criteria	5	Specify the inclusion and exclusion criteria for the review and how studies were grouped for the syntheses.	4
Information sources	6	Specify all databases, registers, websites, organisations, reference lists and other sources searched or consulted to identify studies. Specify the date when each source was last searched or consulted.	4
Search strategy	7	Present the full search strategies for all databases, registers and websites, including any filters and limits used.	Supplemental Information
Selection process	8	Specify the methods used to decide whether a study met the inclusion criteria of the review, including how many reviewers screened each record and each report retrieved, whether they worked independently, and if applicable, details of automation tools used in the process.	4
Data collection process	9	Specify the methods used to collect data from reports, including how many reviewers collected data from each report, whether they worked independently, any processes for obtaining or confirming data from study investigators, and if applicable, details of automation tools used in the process.	4
Data items	10a	List and define all outcomes for which data were sought. Specify whether all results that were compatible with each outcome domain in each study were sought (e.g. for all measures, time points, analyses), and if not, the methods used to decide which results to collect.	4-5
	10b	List and define all other variables for which data were sought (e.g. participant and intervention characteristics, funding sources). Describe any assumptions made about any missing or unclear information.	4-5

3. Section and Topic	Item #	Checklist item	Location where item is reported
Study risk of bias assessment	11	Specify the methods used to assess risk of bias in the included studies, including details of the tool(s) used, how many reviewers assessed each study and whether they worked independently, and if applicable, details of automation tools used in the process.	5
Effect measures	12	Specify for each outcome the effect measure(s) (e.g. risk ratio, mean difference) used in the synthesis or presentation of results.	5
Synthesis methods	13a	Describe the processes used to decide which studies were eligible for each synthesis (e.g. tabulating the study intervention characteristics and comparing against the planned groups for each synthesis (item #5)).	N/A
	13b	Describe any methods required to prepare the data for presentation or synthesis, such as handling of missing summary statistics, or data conversions.	6
	13c	Describe any methods used to tabulate or visually display results of individual studies and syntheses.	5-6
	13d	Describe any methods used to synthesize results and provide a rationale for the choice(s). If meta-analysis was performed, describe the model(s), method(s) to identify the presence and extent of statistical heterogeneity, and software package(s) used.	5-6
	13e	Describe any methods used to explore possible causes of heterogeneity among study results (e.g. subgroup analysis, meta-regression).	5
	13f	Describe any sensitivity analyses conducted to assess robustness of the synthesized results.	5
Reporting bias assessment	14	Describe any methods used to assess risk of bias due to missing results in a synthesis (arising from reporting biases).	5
Certainty assessment	15	Describe any methods used to assess certainty (or confidence) in the body of evidence for an outcome.	5
**RESULTS**			
Study selection	16a	Describe the results of the search and selection process, from the number of records identified in the search to the number of studies included in the review, ideally using a flow diagram.	6
	16b	Cite studies that might appear to meet the inclusion criteria, but which were excluded, and explain why they were excluded.	6, Figure 1
Study characteristics	17	Cite each included study and present its characteristics.	6, Table 1-3
Risk of bias in	18	Present assessments of risk of bias for each included study.	8,

3. Section and Topic	Item #	Checklist item	Location where item is reported
studies			Supplemental Information
Results of individual studies	19	For all outcomes, present, for each study: (a) summary statistics for each group (where appropriate) and (b) an effect estimate and its precision (e.g. confidence/credible interval), ideally using structured tables or plots.	Table 4-6, Supplemental Information
Results of syntheses	20a	For each synthesis, briefly summarise the characteristics and risk of bias among contributing studies.	8-9, Supplemental Information
	20b	Present results of all statistical syntheses conducted. If meta-analysis was done, present for each the summary estimate and its precision (e.g. confidence/credible interval) and measures of statistical heterogeneity. If comparing groups, describe the direction of the effect.	8-9, Table 4-6, Supplemental Information
	20c	Present results of all investigations of possible causes of heterogeneity among study results.	8-9, Table 4-6, Supplemental Information
	20d	Present results of all sensitivity analyses conducted to assess the robustness of the synthesized results.	8-9, Table 4-6, Supplemental Information
Reporting biases	21	Present assessments of risk of bias due to missing results (arising from reporting biases) for each synthesis assessed.	8-9, Supplemental Information
Certainty of evidence	22	Present assessments of certainty (or confidence) in the body of evidence for each outcome assessed.	8-9, Supplemental Information
**DISCUSSION**			
Discussion	23a	Provide a general interpretation of the results in the context of other evidence.	10-11
	23b	Discuss any limitations of the evidence included in the review.	10-11
	23c	Discuss any limitations of the review processes used.	10-11
	23d	Discuss implications of the results for practice, policy, and future research.	10-12
**OTHER INFORMATION**			
NA	24a	Provide registration information for the review, including register name and registration number, or state that the review was not registered.	NA
	24b	Indicate where the review protocol can be accessed, or state that a	NA

3. Section and Topic	Item #	Checklist item	Location where item is reported
		protocol was not prepared.	
	24c	Describe and explain any amendments to information provided at registration or in the protocol.	NA
Support	25	Describe sources of financial or non-financial support for the review, and the role of the funders or sponsors in the review.	NA
Competing interests	26	Declare any competing interests of review authors.	NA
Availability of data, code and other materials	27	Report which of the following are publicly available and where they can be found: template data collection forms; data extracted from included studies; data used for all analyses; analytic code; any other materials used in the review.	12, Supplemental Information

From: [1] Page MJ, McKenzie JE, Bossuyt PM, Boutron I, Hoffmann TC, Mulrow CD, et al. The PRISMA 2020 statement: an updated guideline for reporting systematic reviews. BMJ 2021;372:n71. doi: 10.1136/bmj.n7

b. Supplemental Table 2: MOOSE Checklist

Item No	Recommendation	Reported on Page No
Reporting of background should include		
1	Problem definition	3
2	Hypothesis statement	NA
3	Description of study outcome(s)	3
4	Type of exposure or intervention used	NA
5	Type of study designs used	3
6	Study population	3
Reporting of search strategy should include		
7	Qualifications of searchers (eg, librarians and investigators)	1
8	Search strategy, including time period included in the synthesis and key words	4 Supplemental Information
9	Effort to include all available studies, including contact with authors	1
10	Databases and registries searched	4, Supplemental Information
11	Search software used, name and version, including special features used (eg, explosion)	4, Supplemental Information
12	Use of hand searching (eg, reference lists of obtained articles)	4, Supplemental Information
13	List of citations located and those excluded, including justification	14-17, Figure 1
14	Method of addressing articles published in languages other than English	NA
15	Method of handling abstracts and unpublished studies	Figure 1
16	Description of any contact with authors	NA
Reporting of methods should include		
17	Description of relevance or appropriateness of studies assembled for assessing the hypothesis to be tested	4-5
18	Rationale for the selection and coding of data (eg, sound clinical principles or convenience)	4-5
19	Documentation of how data were classified and coded (eg, multiple raters, blinding and interrater reliability)	4-5
20	Assessment of confounding (eg, comparability of cases and controls in studies where appropriate)	NA

21	Assessment of study quality, including blinding of quality assessors, stratification or regression on possible predictors of study results	4-5
22	Assessment of heterogeneity	Figure 4-6, Supplemental Information
23	Description of statistical methods (eg, complete description of fixed or random effects models, justification of whether the chosen models account for predictors of study results, dose-response models, or cumulative meta-analysis) in sufficient detail to be replicated	NA
24	Provision of appropriate tables and graphics	Tables 1-6, Figures 1-4
Reporting of results should include		
25	Graphic summarizing individual study estimates and overall estimate	Figures 2-4, Supplemental Information
26	Table giving descriptive information for each study included	Table 1-3
27	Results of sensitivity testing (eg, subgroup analysis)	NA
28	Indication of statistical uncertainty of findings	12-16
**Item No**	**Recommendation**	**Reported on Page No**
Reporting of discussion should include		
29	Quantitative assessment of bias (eg, publication bias)	Supplemental Information
30	Justification for exclusion (eg, exclusion of non-English language citations)	4, Figure 1
31	Assessment of quality of included studies	5, Supplemental Information
Reporting of conclusions should include		
32	Consideration of alternative explanations for observed results	11-12
33	Generalization of the conclusions (ie, appropriate for the data presented and within the domain of the literature review)	11-12
34	Guidelines for future research	11-12
35	Disclosure of funding source	13

From: Stroup DF, Berlin JA, Morton SC, et al, for the Meta-analysis Of Observational Studies in Epidemiology (MOOSE) Group. Meta-analysis of Observational Studies in Epidemiology. A Proposal for Reporting. JAMA. 2000;283(15):2008-2012. doi: 10.1001/jama.283.15.2008.

c. Supplemental Table 3: Modified Jadad Analysis for Methodological Quality

StudyID	Authors	Criteria 1	Criteria 2	Criteria 3	Criteria 4	Criteria 5	Criteria 6	Criteria 7	Criteria 8	Total
1	Agbonon et al.	1	0	0	0	0	1	1	1	4
2	Akhtar et al.	0	0	0.5	1	0	1	0	1	3.5
3	Bai et al.	0	0	0.5	1	0	1	1	1	4.5
4	Bao et al.	0	0	0.5	1	0	1	0	1	3.5
5	Braemswig et al.	0	0	0.5	1	0	1	1	1	4.5
6	Brauner et al.	1	1	0.5	1	0	1	1	1	6.5
7	Brundel et al.	0	0	0.5	1	1	1	0	1	4.5
8	Capuana et al.	0	0	0	0	0	1	1	1	3
9	Chacon-Portillo et al.	0	0	0.5	1	0	1	1	1	4.5
10	Chen et al.	0	0	0.5	1	0	1	0	1	3.5
11	Choi et al.	0	0	0.5	1	0	1	1	1	4.5
12	Dannenberg et al.	0	0	0	0	0	1	1	1	3
13	Dassan et al.	0	0	0.5	1	0	1	0	1	3.5
14	Derraz et al.	0	0	0.5	1	0	1	1	1	4.5
15	Diker et al.	0	0	0.5	1	0	1	0	1	3.5

16	Elnekeidy et al.	0	0	0	0	0	1	0	0	1
17	Fan et al.	0	0	0	0	0	1	1	1	3
18	Fiehler et al.	0	0	0.5	1	0	1	1	1	4.5
19	Gao et al.	0	0	0.5	1	0	1	0	1	3.5
20	Gratz et al.	0	0	0.5	1	0	1	1	1	4.5
21	Gregoire et al.	0	0	0.5	1	0	1	0	1	3.5
22	Guo et al.	0	0	0.5	1	0	1	0	1	3.5
23	Han et al.	0	0	0.5	1	0	1	0	1	3.5
24	Horstmann et al.	0	0	0.5	1	0	1	0	1	3.5
25	Hou et al.	0	0	0	0	0	1	0	1	2
26	Huang et al.	0	0	0.5	1	1	1	0	1	4.5
27	Jablonski et al.	0	0	0.5	1	0	1	1	1	4.5
28	Jeon et al.	0	0	0.5	1	0	1	0	1	3.5
29	Kakuda et al.	0	0	0.5	1	0	1	1	1	4.5
30	Kato et al.	0	0	0	0	0	1	0	1	2
31	Khaladkar et al.	0	0	0	0	0	0	0	0	0
32	Kidwell et al.	0	0	0.5	1	0	1	1	1	4.5
33	Ho et al.	0	0	0.5	1	0	1	1	1	4.5

34	Kimura et al.	0	0	0.5	1	0	1	1	1	4.5
35	Lau et al.	0	0	0.5	1	1	1	1	1	5.5
36	Lau et al.	0	0	0.5	1	1	1	1	1	5.5
37	Lee et al.	0	0	0.5	1	0	1	1	1	4.5
38	Lee et al.	0	0	0.5	1	0	1	1	1	4.5
39	Li et al.	0	0	0.5	1	0	1	1	1	4.5
40	Liang et al.	0	0	0.5	1	1	1	1	1	5.5
41	Liu et al.	0	0	0.5	1	1	1	1	1	5.5
42	Luo et al.	0	0	0.5	1	0	1	0	1	3.5
43	Moriya et al.	0	0	0	0	0	1	1	1	3
44	Nagaraja et al.	0	0	0.5	1	0	1	1	1	4,5
45	Nagaraja et al.	0	0	0	0	0	1	1	1	3
46	Naka et al.	0	0	0.5	1	0	1	0	1	3.5
47	Naka et al.	0	0	0.5	0	0	1	0	1	3.5
48	Naka et al.	0	0	0.5	1	0	1	0	1	3.5
49	Nam et al.	0	0	0.5	1	0	1	0	1	3.5
50	Nasreldein et al.	0	0	0.5	1	1	1	0	1	4.5
51	Nighoghossian et al.	0	0	0.5	1	0	1	1	1	4.5
52	Orken et al.	0	0	0	0	0	1	0	1	2

53	Ozbek et al.	0	0	0	0	0	1	1	1	3
54	Potigumjon et al.	0	0	0	0	0	1	1	1	3
55	Purrucker et al.	0	0	0.5	1	1	1	1	1	5.5
56	Ryu et al.	0	0	0.5	1	0	1	0	1	3.5
57	Schlemm et al.	1	1	1	1	0	1	1	1	7
58	Shahjouei et al.	0	0	0	0	0	1	0	1	2
59	Shi et al.	0	0	0.5	1	0	1	1	1	4.5
60	Soo et al.	0	0	0	0	0	1	1	1	3
61	Soo et al.	0	0	0	0	1	1	1	1	4
62	Sun et al.	0	0	0	0	0	1	1	1	3
63	Takahashi et al.	0	0	0.5	1	0	1	0	1	3.5
64	Takahashi et al.	0	0	0	0	0	1	1	1	3
65	Turc et al.	0	0	0.5	1	0	1	1	1	4.5
66	Wang et al.	0	0	0	0	0	1	1	1	3
67	Wang et al.	0	0	0.5	1	0	1	1	1	4.5
68	Wang et al.	0	0	0.5	1	0	1	1	1	4.5

69	Werring et al.	0	0	0.5	1	0	1	0	1	3.5
70	Xu et al.	0	0	0	0	0	1	1	1	3
71	Yan et al.	0	0	0.5	1	0	1	1	1	4.5
72	Yan et al.	0	0	0	0	0	1	1	1	3
73	Yang et al.	0	0	0.5	1	0	1	1	1	4.5
74	Zand et al.	0	0	0	0	0	1	1	1	3
75	Zand et al.	0	0	0.5	1	0	1	1	1	4.5
76	Zhang et al.	0	0	0.5	1	0	1	0	1	3.5
77	Zhang et al.	0	0	0.5	1	0	1	0	1	3.5
78	Zhao et al.	0	0	0	0	0	1	1	1	3
79	Zhao et al.	0	0	0	0	0	1	1	1	3
80	Zhao et al.	0	0	0.5	1	0	1	1	1	4.5

Criteria 1: Was the study randomised? (0 = not described or no, 1 = yes)

Criteria 2: Was the method of randomisation appropriate (0 = not described or no, 1 = yes)

Criteria 3: Was the study described as being blinded? (0 = not described or no, 0.5 = single blinded 1 = double-blinded)

Criteria 4: Was the method of blinding appropriate (0 = not described or no, 1 = yes)

Criteria 5: Was there a description of withdrawals and dropouts? (0 = not described or no, 1 = yes)

Criteria 6: Was there a clear description of the inclusion/exclusion criteria? (0 = not described or no, 1 = yes)

Criteria 7: Was the method used to assess adverse events described? (0 = not described or no, 1 = yes)

Criteria 8: Was the method of statistical analysis described? (0 = not described or no, 1 = yes

d. Supplemental Table 4: Funding Bias Scores for Studies

StudyID	Authors	Publication Bias	Funding
1	Agbonon et al.	0	No conflicts of interest
2	Akhtar et al.	0	No conflicts of interest
3	Bai et al.	0	Supported by the Key Discipline Construction Project of the Pudong Health Bureau of Shanghai, Outstanding Leader Training Program of the Pudong Health Bureau of Shanghai, , Research Grant for Health Science and Technology of the Pudong Health Bureau of Shanghai, , and the National Eleven-Fifth Scientific and Technological Brainstorm project.
4	Bao et al.	0	No conflicts of interest
5	Braemswig et al.	0	No conflicts of interest
6	Brauner et al.	0	Sponsored by the Foundation A. de Rothschild and funded by the French Health Ministry
7	Brundel et al.	0	No conflicts of interest
8	Capuana et al.	0	No conflicts of interest
9	Chacon-Portillo et al.	0	No conflicts of interest
10	Chen et al.	0	No conflicts of interest
11	Choi et al.	0	No conflicts of interest
12	Dannenberg et al.	0	No conflicts of interest

13	Dassan et al.	0	Supported by a project grant from the Stroke Association and by funding from the Department of Health's National Institute for Health Research Centre funding scheme.
14	Derraz et al.	0	No conflicts of interest
15	Diker et al.	0	No conflicts of interest
16	Elnekeidy et al.	0	No conflicts of interest
17	Fan et al.	0	Supported by the Neurology/Angiology Fund of the Chinese University of Hong Kong
18	Fiehler et al.	1	Primary author received speaker fees from Bracco ALTANA
19	Gao et al.	0	No conflicts of interest
20	Gratz et al.	0	An author is supported by the Swiss National Science Foundation
21	Gregoire et al.	0	Supported by the Department of Health's National Institute for Health Research Biomedical Research Centres and the Stroke Association
22	Guo et al.	0	Supported by the Nature Science Fundation of Hebei Province, Scientific and Technological Innovation 2030-Major Project Subject of Brain Science and Brain-inspired Research, and Hebei Province Government-funded Excellent Talents Project in Clinical Medicine
23	Han et al.	N/A	No conflicts of interest declared
24	Horstmann et al.	1	An author has received consulting honoraria, research support, travel grants and speakers' honoraria from Bayer HealthCare, Boehringer Ingelheim, BMS Pfizer, Roche Diagnostics, St Jude Medical and Sanofi Aventis
25	Hou et al.	0	No conflicts of interest
26	Huang et al.	0	No conflicts of interest

27	Jablonski et al.	2	Received funding from Siemens Heathineers.
28	Jeon et al.	0	Supported by a grant from the Brain Research Center of the 21st Century Frontier Research Program funded by the Ministry of Science and Technology of Korea
29	Kakuda et al.	1	NIH funded this entire study, except for the tPA, which was provided by Genentech at no charge. Some authors have consulting relationships with Genentech
30	Kato et al.	N/A	No conflicts of interest declared
31	Khaladkar et al.	0	No conflicts of interest
32	Kidwell et al.	0	Supported by grants from the American Heart Association, National Stroke Association, and the National Institute of Neurological Disorders and Stroke
33	Ho et al.	0	Supported by the Korean Ministry of National Health and Welfare
34	Kimura et al.	1	Primary author receives honoraria from Mitsubishi-Tanabe Pharma, Boehringer Ingelheim, Bayer AG, and Otsuka Pharmaceutical Co, Ltd.
35	Lau et al.	0	No conflicts of interest
36	Lau et al.	0	Funded by the National Institute for Health Research Oxford Biomedical Research Centre
37	Lee et al.	N/A	No conflicts of interest declared
38	Lee et al.	0	No conflicts of interest
39	Li et al.	0	No conflicts of interest
40	Liang et al.	0	Supported by the National Natural Science Foundation of China
41	Liu et al.	0	No conflicts of interest

42	Luo et al.	0	Supported by the National Natural Science Foundation and the Research Fund of Shanghai Fourth People's Hospital
43	Moriya et al.	N/A	No conflicts of interest declared
44	Nagaraja et al.	1	Primary author is a consultant stroke adjudicator for the Women's Health Initiative study and receives research funding from the 1Florida Alzheimer's Disease Research Center
45	Nagaraja et al.	N/A	No conflicts of interest declared
46	Naka et al.	N/A	No conflicts of interest declared
47	Naka et al.	N/A	No conflicts of interest declared
48	Naka et al.	N/A	No conflicts of interest declared
49	Nam et al.	0	No conflicts of interest
50	Nasreldein et al.	0	No conflicts of interest
51	Nighoghossian et al.	0	Supported by the HCL Research Office and Siemens Society, and funded by ARNEI
52	Orken et al.	0	No conflicts of interest
53	Ozbek et al.	0	No conflicts of interest
54	Potigumjon et al.	0	No conflicts of interest
55	Purrucker et al.	1	Primary author received travel and congress participation support from Pfizer, and personal fees from Boehringer Ingelheim
56	Ryu et al.	0	No conflicts of interest
57	Schlemm et al.	1	Primary author is a participant in the Berlin Institute of Health-Charité Clinical Scientist Program funded by the Charité-Universitätsmedizin Berlin and the Berlin

			Institute of Health and reports lecture fees from Daiichi Sankyo, outside of the submitted work
58	Shahjouei et al.	0	No conflicts of interest
59	Shi et al.	1	Supported by NIH/NINDS. Authors have consultancy roles with Stryker, Covidien, CoAxia, and more
60	Soo et al.	0	Funded by S. H. Ho Foundation, Vascular and Interventional Radiology Foundation, and Department of Medicine and Therapeutics, Prince of Wales Hospital, The Chinese University of Hong Kong.
61	Soo et al.	0	No conflicts of interest
62	Sun et al.	0	Supported by Sun Yatsen University
63	Takahashi et al.	0	Supported by Intramural Research Fund for Cardiovascular Diseases of National Cerebral and Cardiovascular Center
64	Takahashi et al.	N/A	No conflicts of interest declared
65	Turc et al.	0	An author is supported by the Societe Francaise de Radiologie
66	Wang et al.	N/A	No conflicts of interest declared
67	Wang et al.	0	No conflicts of interest
68	Wang et al.	0	No conflicts of interest
69	Werring et al.	0	No conflicts of interest
70	Xu et al.	0	No conflicts of interest
71	Yan et al.	1	An author is a consultant/advisory board member for Stryker and Covidien
72	Yan et al.	0	No conflicts of interest

73	Yang et al.	0	No conflicts of interest
74	Zand et al.	N/A	No conflicts of interest declared
75	Zand et al.	N/A	No conflicts of interest declared
76	Zhang et al.	0	No conflicts of interest
77	Zhang et al.	0	Study was funded by the Ministry of Science and Technology and the Ministry of Health of the People's Republic of China
78	Zhao et al.	0	No conflicts of interest
79	Zhao et al.	N/A	No conflicts of interest declared
80	Zhao et al.	0	No conflicts of interest

0 = Low potential for bias

1 = Conflicts of interest declared relating to industry funding outside of current research publication

2 = Funded by industry

3 = High potential for bias

e. Supplemental Table 5: Outputs from Egger's Test for Publication Bias for Association Variables

Outcome	Std_Eff	Coefficient [95% CI]	Standard Error	t	P > | t |	Test of H0: no small-study effects
sICH	Slope	0.61 [-0.284; 1.500]	0.41	1.49	0.163	0.656
	Bias	0.36 [-1.360; 2.080]	0.79	0.46	0.656	
HT	Slope	0.50 [-0.165; 1.022]	0.25	2.03	0.057	1.488
	Bias	-0.42 [-1.802; 0.957]	0.66	-0.64	0.529	
mRS 3-6 at 90 days	Slope	0.17 [-0.510; 0.850]	0.31	0.56	0.590	1.024
	Bias	1.29 [-1.366; 3.950]	1.19	1.08	0.304	

Abbreviations: sICH = symptomatic intracranial haemorrhage, HT = haemorrhagic transformation, mRS = Modified Rankin Scale, Std_Eff = standardised effect size, CI = confidence interval, t = t-statistic, P = p-value, H0 = null hypothesis.

f. Supplemental Table 6: Outputs from Deeks' Test for Small Study Effects and Publication Bias for Association Variables

Outcome	yb	Coefficient [95% CI]	Standard Error	t	P > | t |
sICH	Bias	-0.46 [-23.444; 22.528]	10.55	-0.04	0.966
	Intercept	0.91 [-0.546; 2.368]	0.67	1.36	0.198
HT	Bias	-2.53 [-13.362; 8.308]	5.18	-0.49	0.631
	Intercept	0.55 [-0.261; 1.366]	0.39	1.42	0.171
mRS 3-6 at 90 days	Bias	4.62 [-16.615; 25.857]	9.53	0.48	0.638
	Intercept	0.26 [-1.005; 1.526]	0.57	0.46	0.656

Abbreviations: sICH = symptomatic intracranial haemorrhage, HT = haemorrhagic transformation, mRS = Modified Rankin Scale, yb = estimated regression coefficient, CI = confidence interval, t = t-statistic, P = p-value.

4. Supplemental Figures

a. Supplemental Figure 1: Forest Plots of CMB Prevalence, Stratified by Age, Hypertension, and Regional Variation

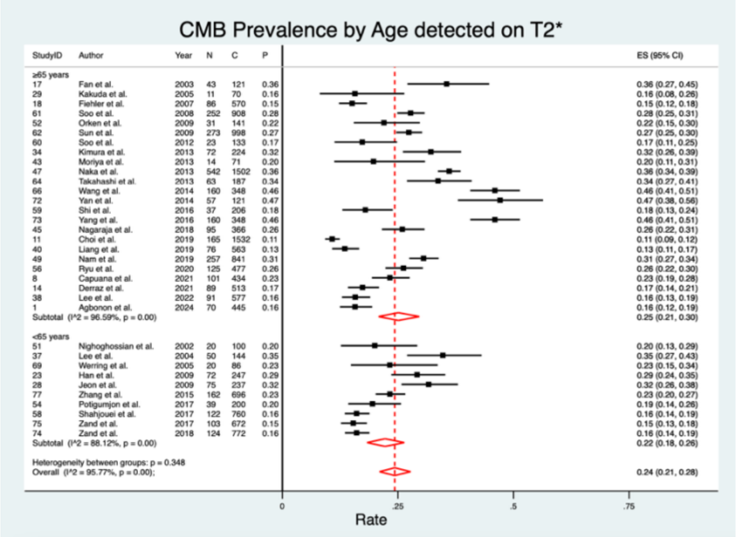

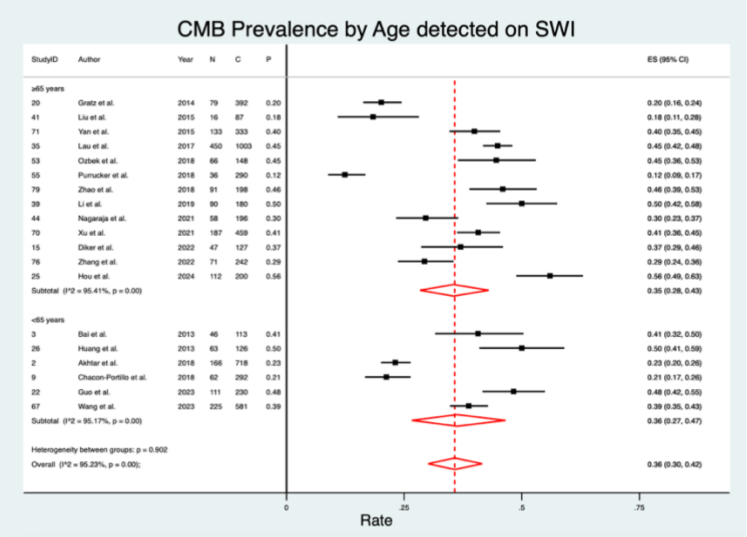

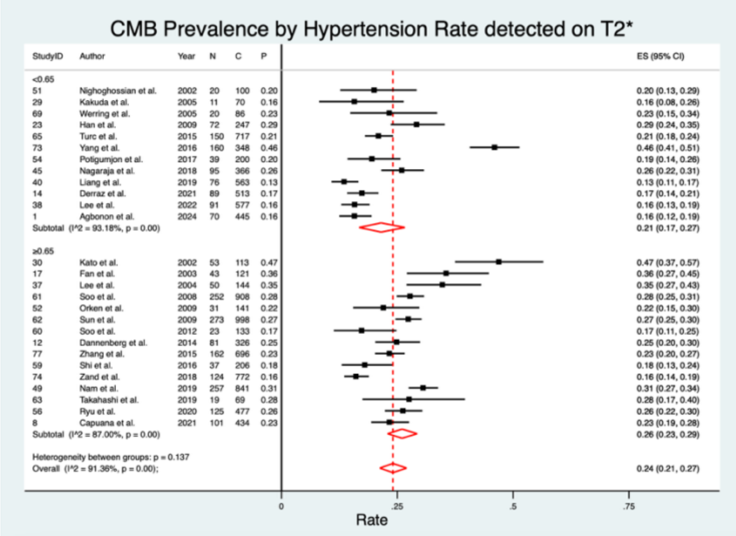

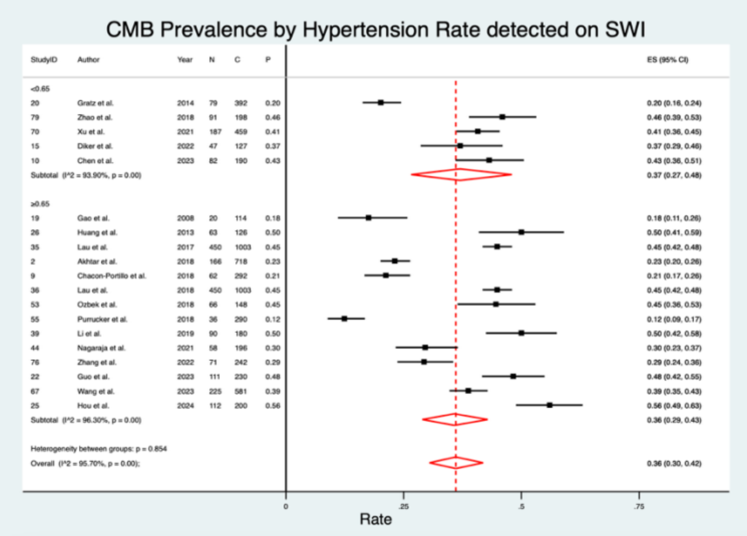

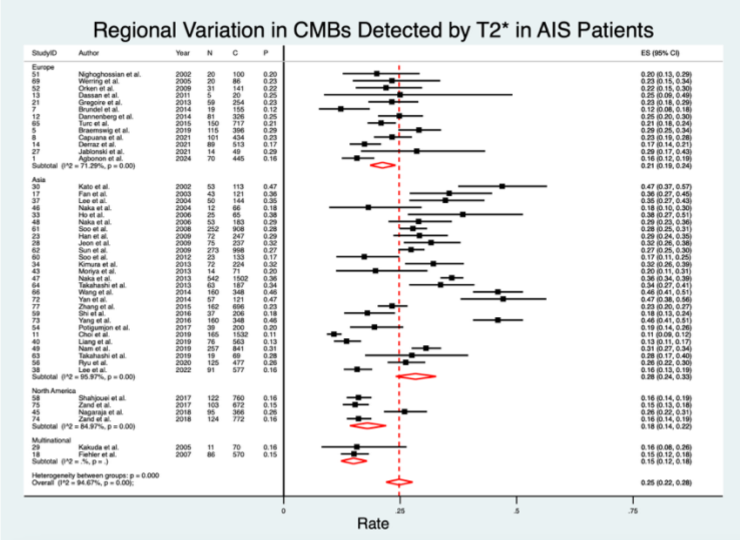

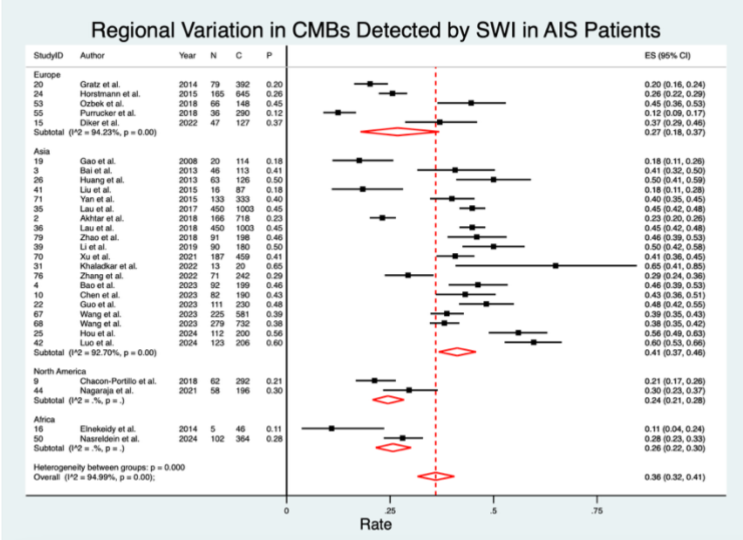

b. Supplemental Figure 2: Forest Plots of CMB Prevalence, Stratified by Use of FLAIR, NCCT, and Slice Thickness

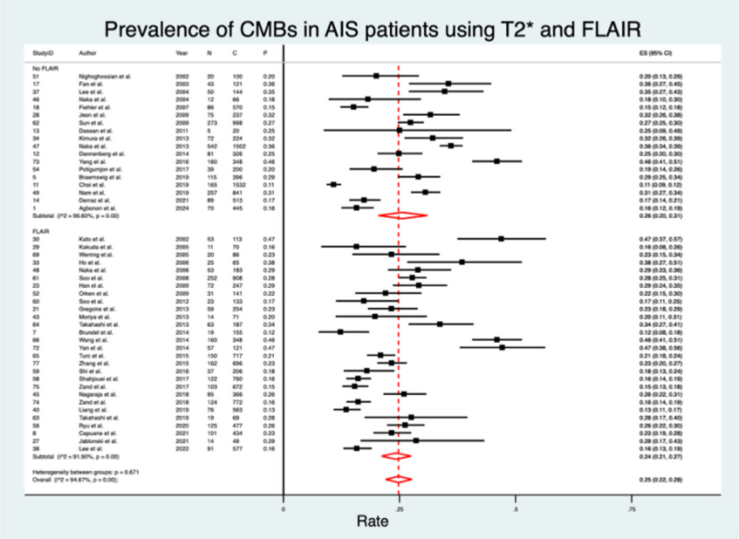

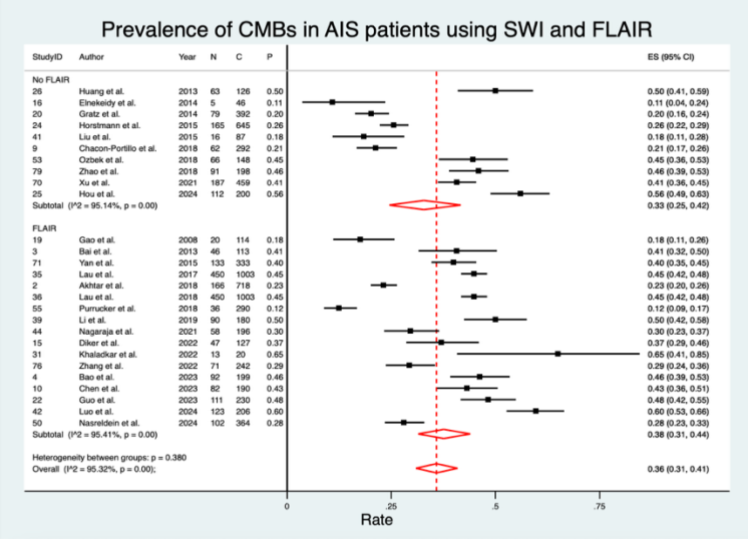

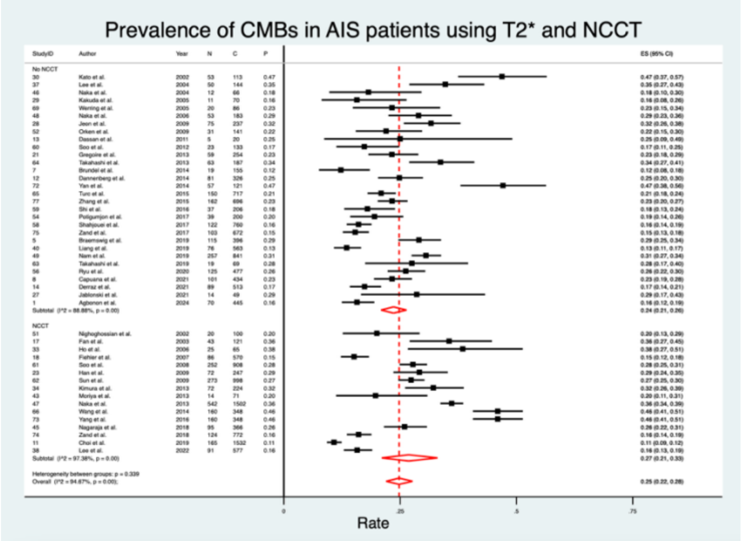

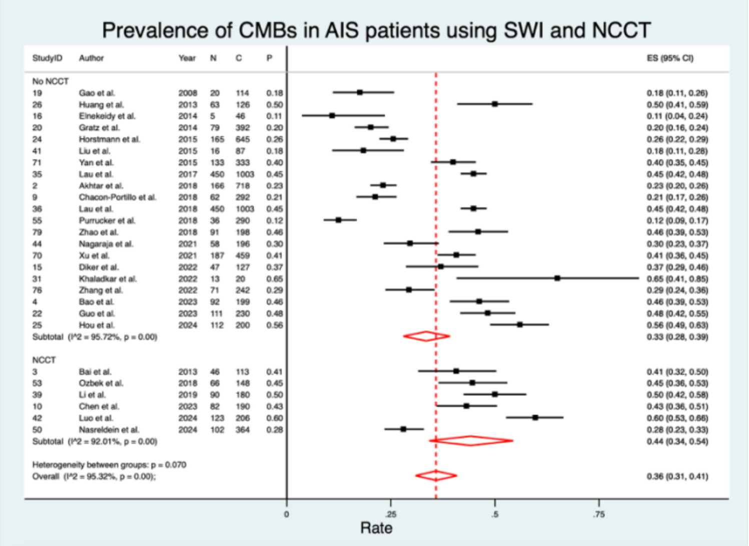

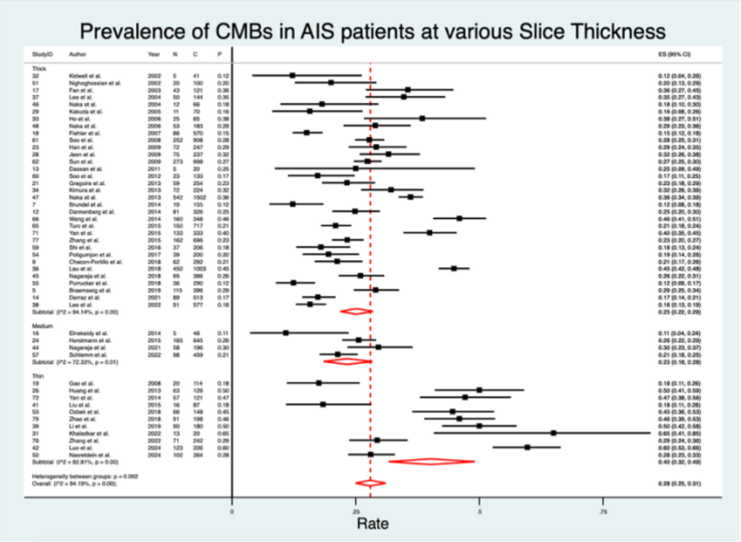

c. Supplemental Figure 3: Forest Plots of CMB Prevalence, Stratified by Field Strength, Stroke Subtype, and CMB Location

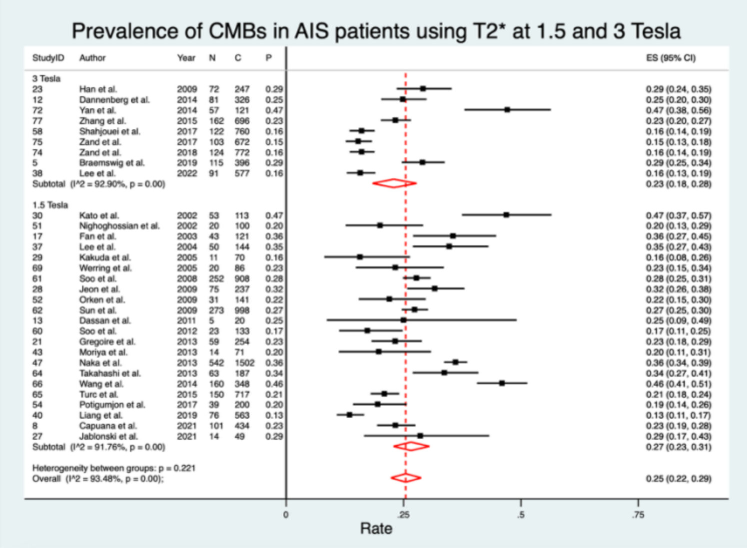

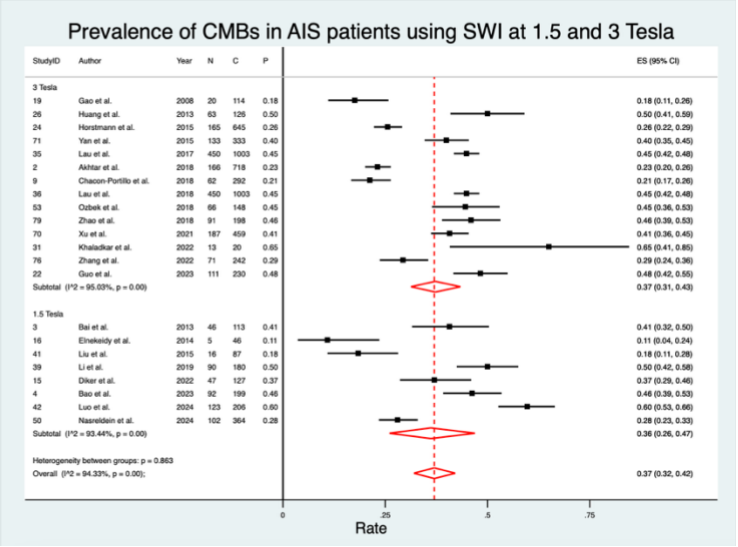

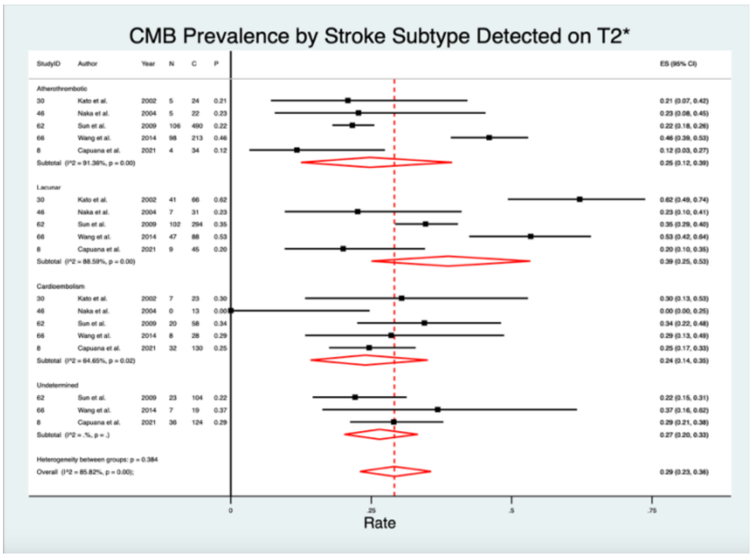

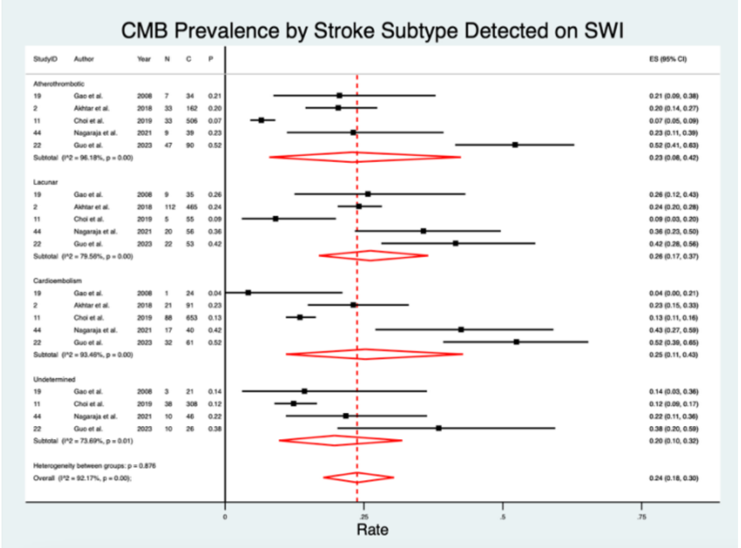

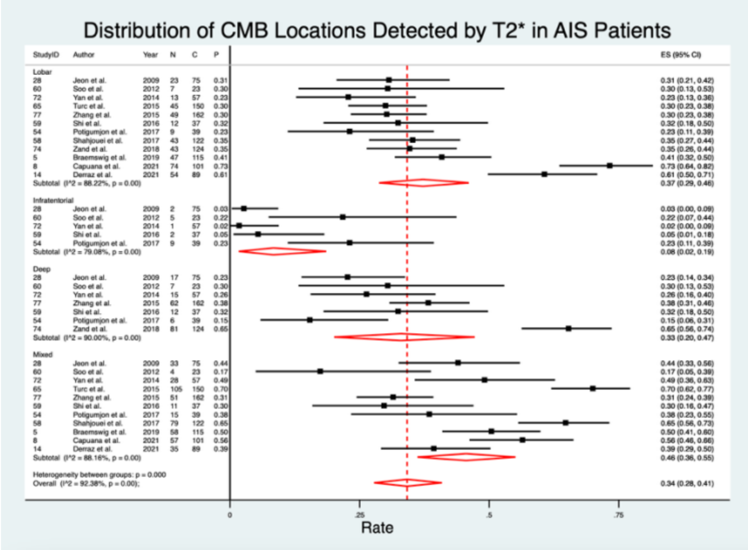

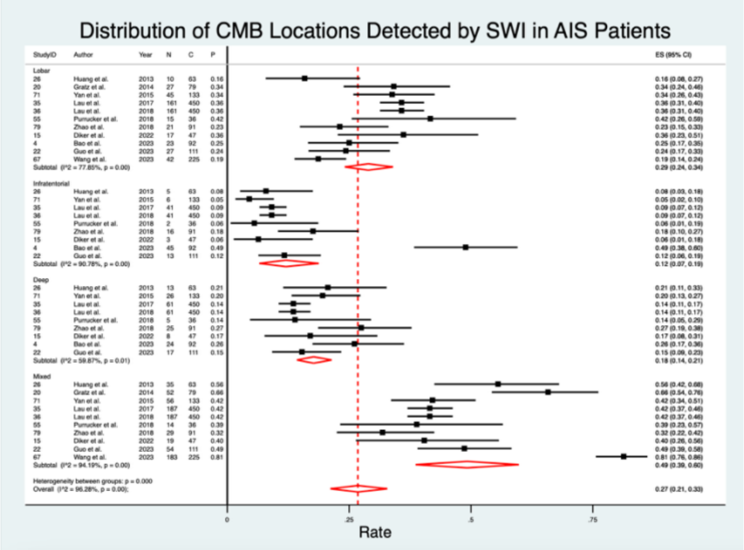

Supplemental Figure 4: Graphs of Egger's Regression Test for Meta-Analysis on the Association between CMBs and Prognostic Outcomes

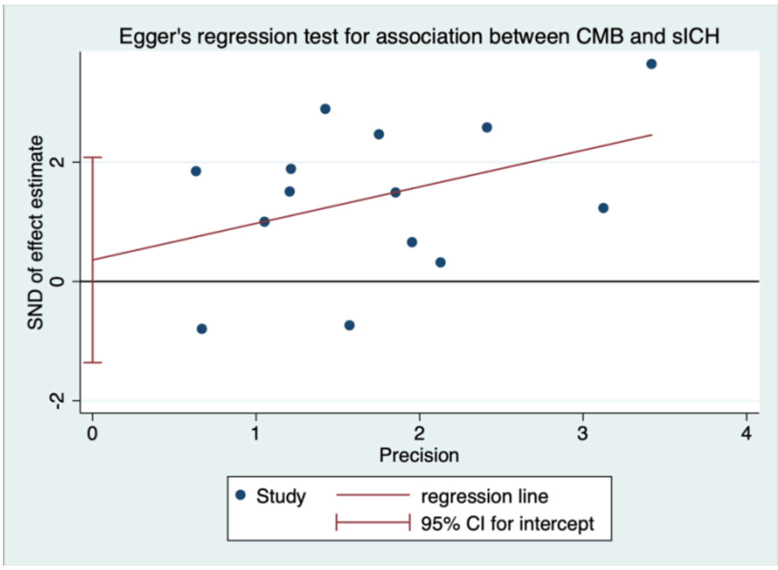

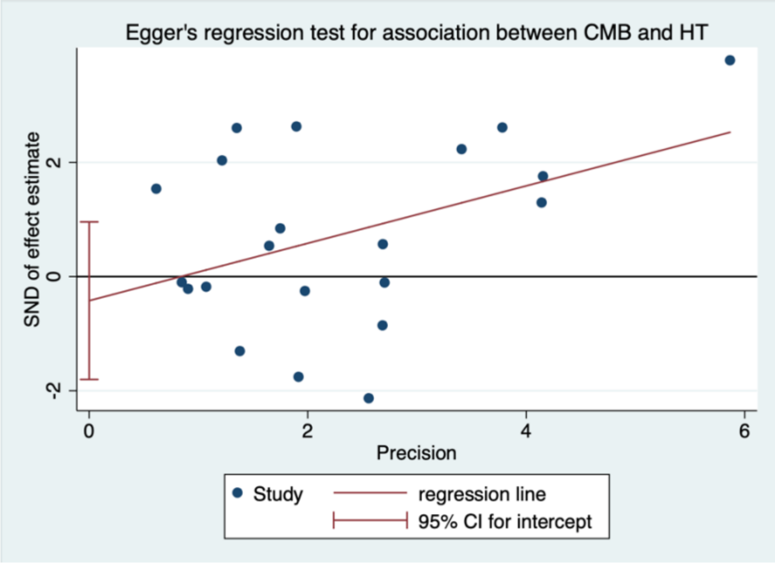

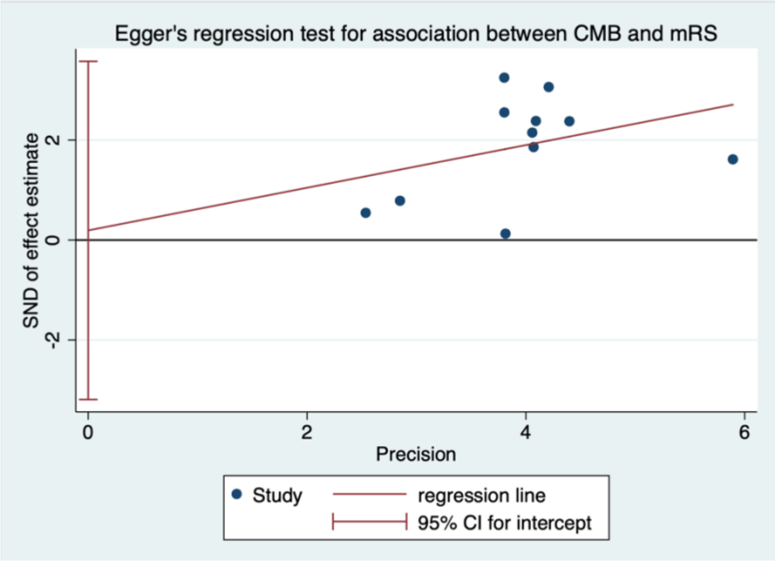

Supplemental Figure 5: Graphs of Funnel Plot for Meta-Analysis on the Association between CMBs and Prognostic Outcomes

<table>
<tr><td>Studies Assessing the Association between CMBs and sICH</td><td>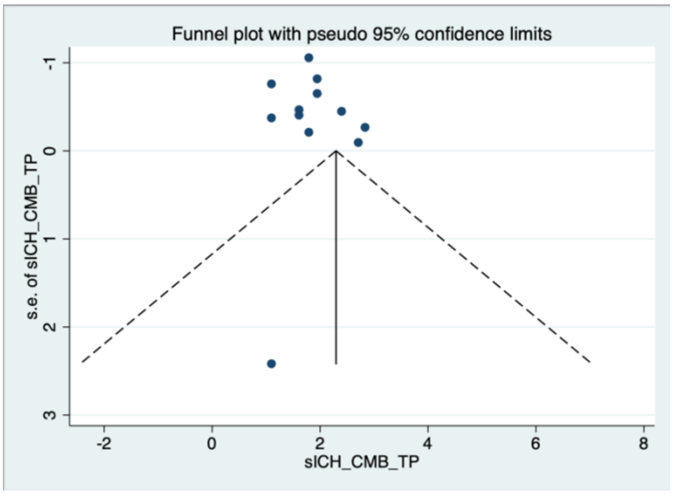
</td></tr>
<tr><td>Studies Assessing the Association between CMBs and HT</td><td>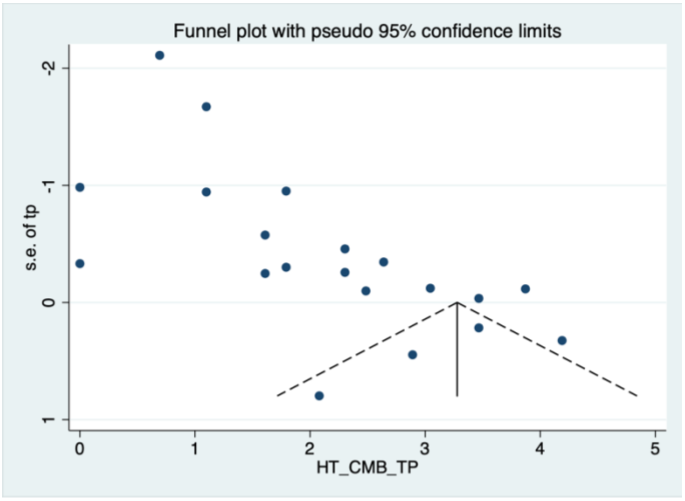
</td></tr>
<tr><td>Studies Assessing the Association between CMBs and mRS scores 3-6</td><td>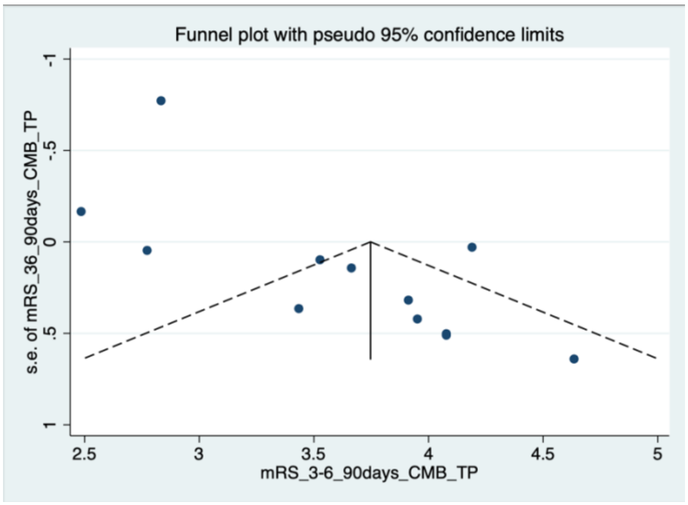
</td></tr>
</table>

Supplemental Figure 6: Sensitivity Analysis on Association between CMBs and Prognostic Outcomes

<table>
<tr>
<td>Studies Assessing the Association between CMBs and sICH</td>
<td>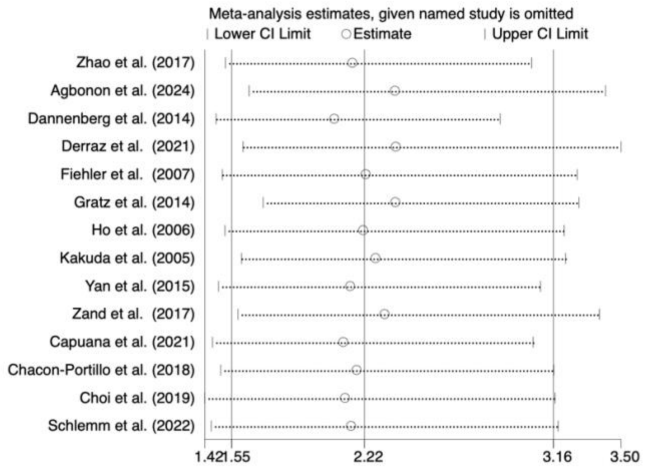
</td>
</tr>
<tr>
<td>Studies Assessing the Association between CMBs and HT</td>
<td>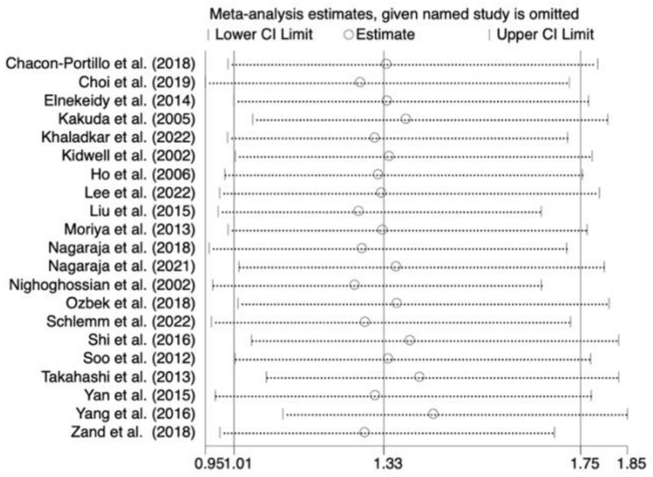
</td>
</tr>
<tr>
<td>Studies Assessing the Association between CMBs and mRS scores 3-6</td>
<td>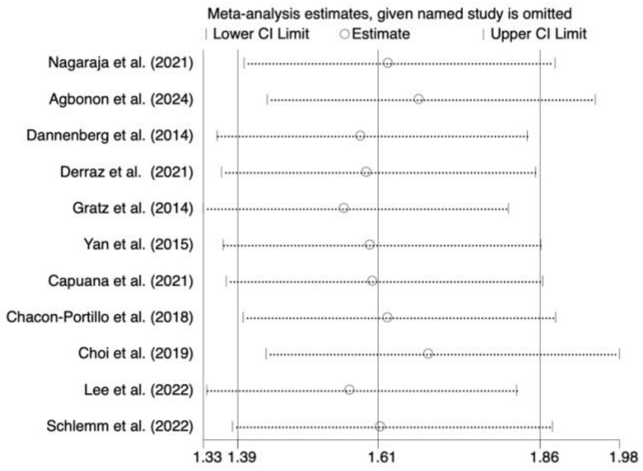
</td>
</tr>
</table>

Supplemental Figure 7: Graphs of ROC Plot for Meta-Analysis on the Association between CMBs and Prognostic Outcomes

<table>
<tr>
<td>Studies Assessing the Association between CMBs and sICH</td>
<td>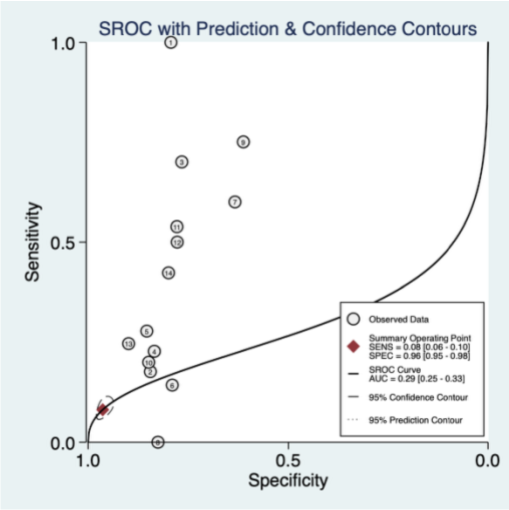
</td>
</tr>
<tr>
<td>Studies Assessing the Association between CMBs and HT</td>
<td>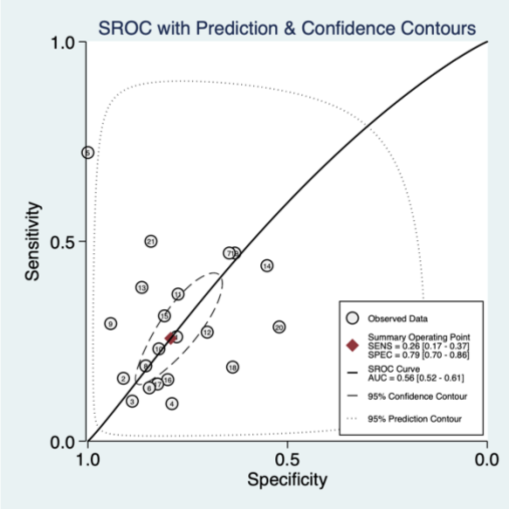
</td>
</tr>
<tr>
<td>Studies Assessing the Association between CMBs and mRS scores 3-6</td>
<td>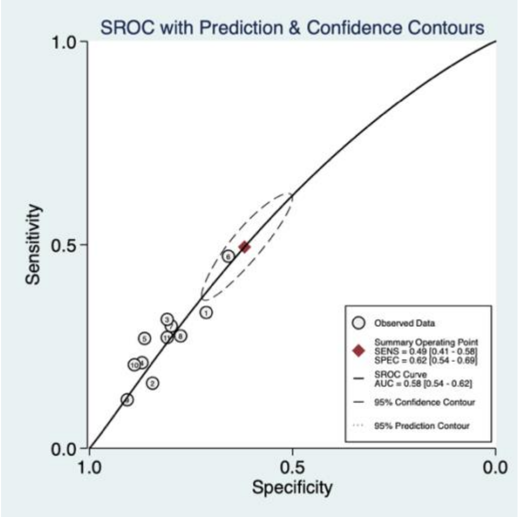
</td>
</tr>
</table>

Supplemental Figure 8: Graphs of Deeks' Funnel Plot for Meta-Analysis on the Association between CMBs and Prognostic Outcomes

Studies Assessing the Association between CMBs and sICH

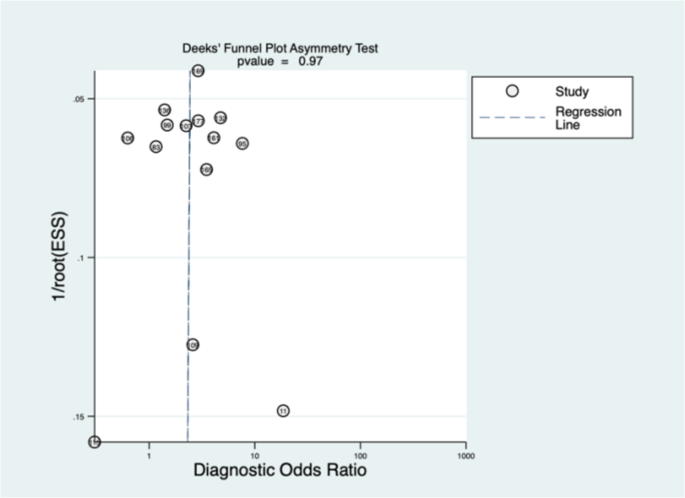

Studies Assessing the Association between CMBs and HT

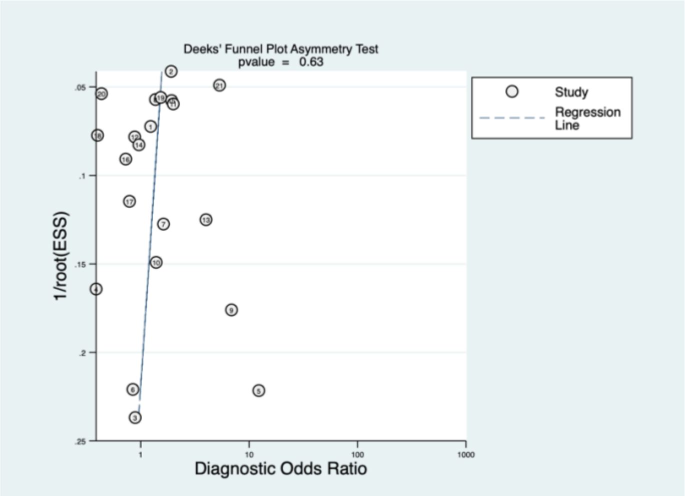

Studies Assessing the Association between CMBs and mRS scores 3-6

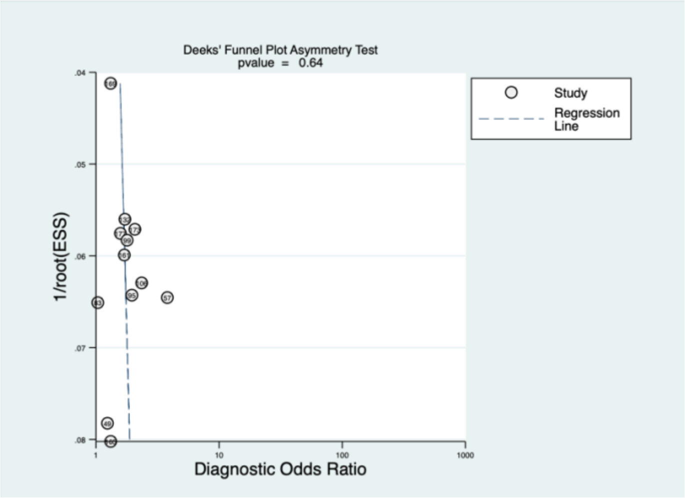

Supplemental Figure 9: Graphs of Fagan's Plot for Meta-Analysis on the Association between CMBs and Prognostic Outcomes

Studies Assessing the Association between CMBs and sICH	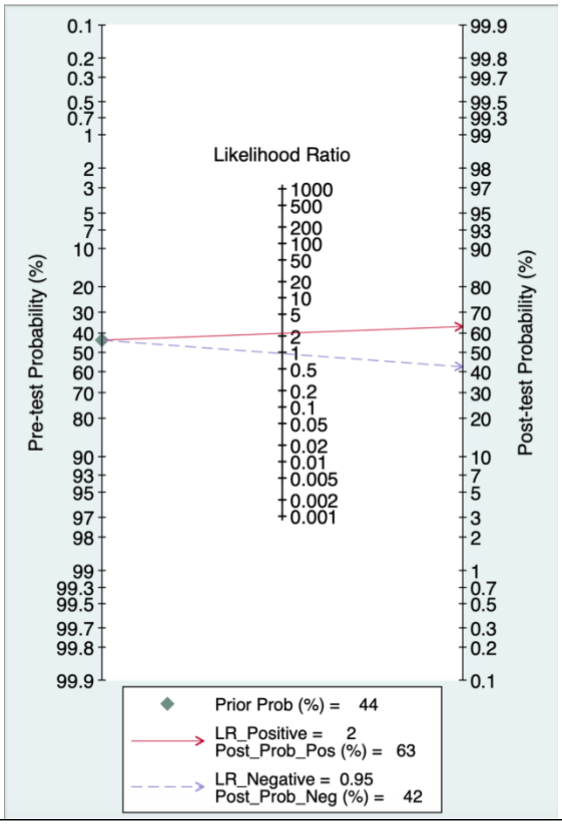
Studies Assessing the Association between CMBs and HT	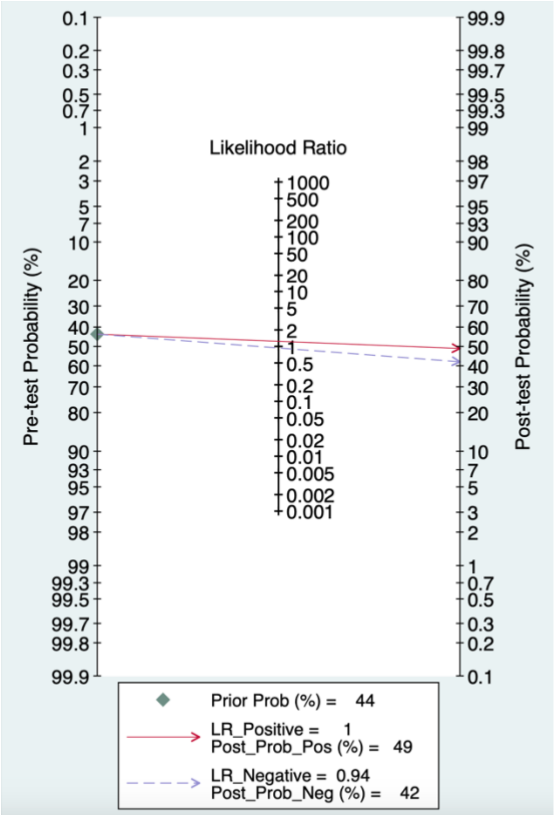

Studies Assessing the Association between CMBs and mRS scores 3-6	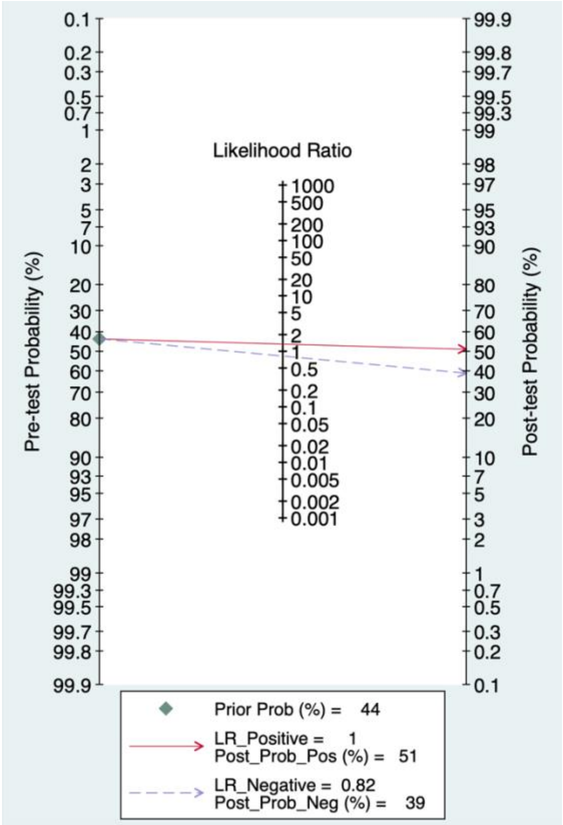
